# Contact Electrification–Based Enantioselective Recognition of Chiral Amino Acids through Stereospecific Interfacial Electron Transfer

**DOI:** 10.1021/jacs.5c15608

**Published:** 2026-01-12

**Authors:** Arnab Pal, Hakjeong Kim, Shreerag Suresh, Po-Han Wei, Abdullah Mohamed Al-Kabbany, Dukhyun Choi, Zong-Hong Lin

**Affiliations:** † Department of Biomedical Engineering, National Taiwan University, Taipei 10617, Taiwan; ‡ School of Mechanical Engineering, College of Engineering, Sungkyunkwan University, Suwon 16419, South Korea

## Abstract

Chirality lies at the heart of chemistry, governing the structure–function relationships of biomolecules, pharmaceuticals, and catalysts. However, rapid and label-free enantioselective analysis remains an enduring challenge due to the intrinsic similarity of enantiomers’ physicochemical properties. Here, we report a contact electrification–based triboelectric sensing platform for the enantioselective recognition of chiral amino acids, achieved by coating CuO nanowires. The approach exploits chirality-dependent interfacial electron transfer, whereby differences in molecular orbital alignment and work function between enantiomers generate distinct electronic signatures during controlled contact–separation with acetone. Kelvin probe force microscopy, ultraviolet photoelectron spectroscopy, and density functional theory calculations reveal that subtle differences in side-chain geometry modulate nanoscale surface potentials and electron cloud overlap, leading to quantifiable shifts in charge transfer efficiency. The method achieves millisecond-scale discrimination across charged, polar uncharged, and sulfur-containing amino acids, with orthogonal evidence from molecule specific enantioselective contact-electrocatalytic degradation of methyl orange. By transducing stereochemical information directly into measurable electrical outputs, this work demonstrates a mechanistically grounded chemical sensing paradigm, offering a versatile platform for pharmaceutical quality control and biomolecular diagnostics.

## Graphical abstract

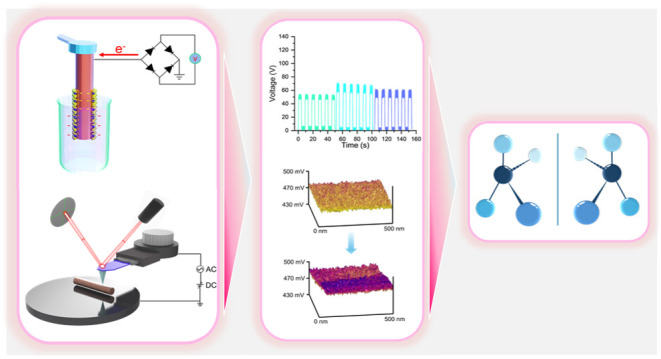

## Introduction

Chirality is a foundational concept in chemistry, shaping the reactivity, binding affinity, and biological function of molecules across the chemical sciences. Enantiomerically pure compounds are essential in pharmaceuticals, agrochemicals, and biomaterials, where even trace amounts of the undesired enantiomer can alter therapeutic efficacy or trigger adverse effects.^1–3^ In biological systems, l-amino acids dominate protein structures, yet d-amino acids—once considered rare—are now recognized as crucial biomarkers for neurological disorders, cancer progression, and renal dysfunction.^4–6^ The reliable recognition of enantiomers is thus both a chemical and biomedical imperative, demanding techniques that combine molecular specificity with operational simplicity.

Conventional chiral analysis relies on spectroscopic and chromatographic strategies, such as circular dichroism spectroscopy and chiral-phase high-performance liquid chromatography, which differentiate enantiomers through small differences in optical absorption or thermodynamic binding energies.^7–9^ While powerful, these methods often require complex instrumentation, lengthy sample preparation, and trained operators, limiting their use in rapid or point-of-care settings. Furthermore, they rarely provide direct insight into the underlying electronic consequences of molecular chirality at solid–liquid interfaces—an aspect that is increasingly recognized as critical in fields ranging from heterogeneous catalysis to molecular electronics.^10–13^

Recent advances in nanoscale interfacial science have opened new pathways for chiral recognition by harnessing fundamental charge transfer phenomena. Contact electrification (CE)—the process by which charge is exchanged between materials during physical contact—has emerged as a versatile, energy-autonomous means of transducing molecular interactions into measurable electrical signals.^14–19^ When combined with high-surface-area semiconductor nanostructures, CE offers a direct link between molecular-level stereochemistry and macroscopic electronic readouts. Crucially, this framework enables the interrogation of chirality-dependent electron transfer processes, which are dictated by the relative alignment of molecular orbitals, the symmetry of electron clouds, and the chemical nature of surface functional groups.^15,20–23^

Here, we present a contact electrification–based triboelectric nanosensor that exploits stereospecific interfacial electron transfer for the enantioselective recognition of chiral amino acids. By coating CuO nanowires^24–28^ with pure L- or d-amino acids, we generate chiral interfaces whose work functions and surface potentials differ subtly yet reproducibly due to variations in molecular geometry and binding orientation.^29,30^ During controlled contact–separation with acetone, these differences manifest as distinct triboelectric voltage outputs, which we correlate with nanoscale surface potential mapping using the Kelvin probe force microscopy, work function determination via ultraviolet photoelectron spectroscopy, and molecular orbital simulations using density functional theory.^31–35^ This approach not only enables rapid, label-free discrimination of amino acids with diverse side-chain chemistries—including charged, polar uncharged, and sulfur-containing groups—but also provides mechanistic insight into how molecular chirality modulates interfacial charge transfer. A comprehensive comparison of chiral molecule detection techniques is represented in Table S1.

By bridging molecular recognition chemistry with interfacial electron transfer physics, this platform establishes a new mechanistic paradigm for chiral sensing. While the current study demonstrates proof-of-concept for three structurally diverse amino acid classes, extension to other chiral molecular families such as pharmaceutical enantiomers or sugars represents a promising future direction that requires substantial adaptation of surface chemistry and rigorous revalidation of the underlying electronic transfer principles. Future works should systematically explore how this mechanistic framework can be adapted for broader applications in pharmaceutical quality control, enantioselective catalysis, and biomolecular diagnostics.

## Result and Discussion

### Stereospecific Contact Electrification Enables Chiral Discrimination of Amino Acids

A comprehensive overview of the contact electrification-based chiral amino acid detection system is depicted in Figure 1. The fundamental structural differences between L- and d-amino acid enantiomers are illustrated, with characteristic mirror-image molecular configurations shown in Figure 1a. The three-dimensional representations clearly demonstrate how identical atomic compositions lead to distinct spatial arrangements of functional groups, forming the basis for chiral discrimination in our detection platform. The triboelectric nanosensor fabrication process is detailed in Figure 1b, depicting the sequential steps of CuO nanowire synthesis through thermal oxidation at 500 °C for 5 h. Then followed by controlled amino acid absorption utilizing the dip-coating method. The scanning electron microscopy (SEM) images reveal the morphological characteristics of the synthesized nanowires. The images display well-defined structures with high aspect ratios and uniform distribution across the substrate surface. Figure 1c presents the proposed mechanism for chiral discrimination through contact electrification, illustrating the electron cloud interactions between amino acids and acetone molecules during contact-separation cycles. This mechanism demonstrates how different molecular systems create varying energy barriers and electron transfer characteristics. Thus, resulting in electrical signatures during the continuous frictional contact separation process as depicted in Figure S1. Moreover, the detailed signal generation via the CE method is explained in Supporting Information S1. The measured voltage output for l/d-aspartic acid and CuO nanowires (Figure 1d) reveals distinct differences, as confirmed by statistical analysis across three independent sample sets (Figure 1e), indicating chirality-dependent voltage differences. Furthermore, the coated CuO surface by the chiral l/d-aspartic acid is investigated using Fourier transform infrared (FTIR) spectroscopy (Figure S2) and SEM images (Figure S3), prior to the CE process. Confirming that the CE occurred between the amino acid-modified surface and the contact solvent. The selection of acetone as the contact solvent was critical for maintaining stable chiral interfaces, as its volatility and inability to dissolve amino acids preserved the molecular architecture required for stereospecific recognition throughout repeated contact-separation cycles. Additionally, acetone is a polar aprotic solvent with minimal ionic conductivity, substantially reducing ion transfer compared to aqueous systems. Hence, triboelectric outputs across amino acid pairs support an electron transfer mechanism. Further discussion about the selection of acetone as a suitable contact solvent is elaborately presented in Supporting Information S2.

**Figure 1:**
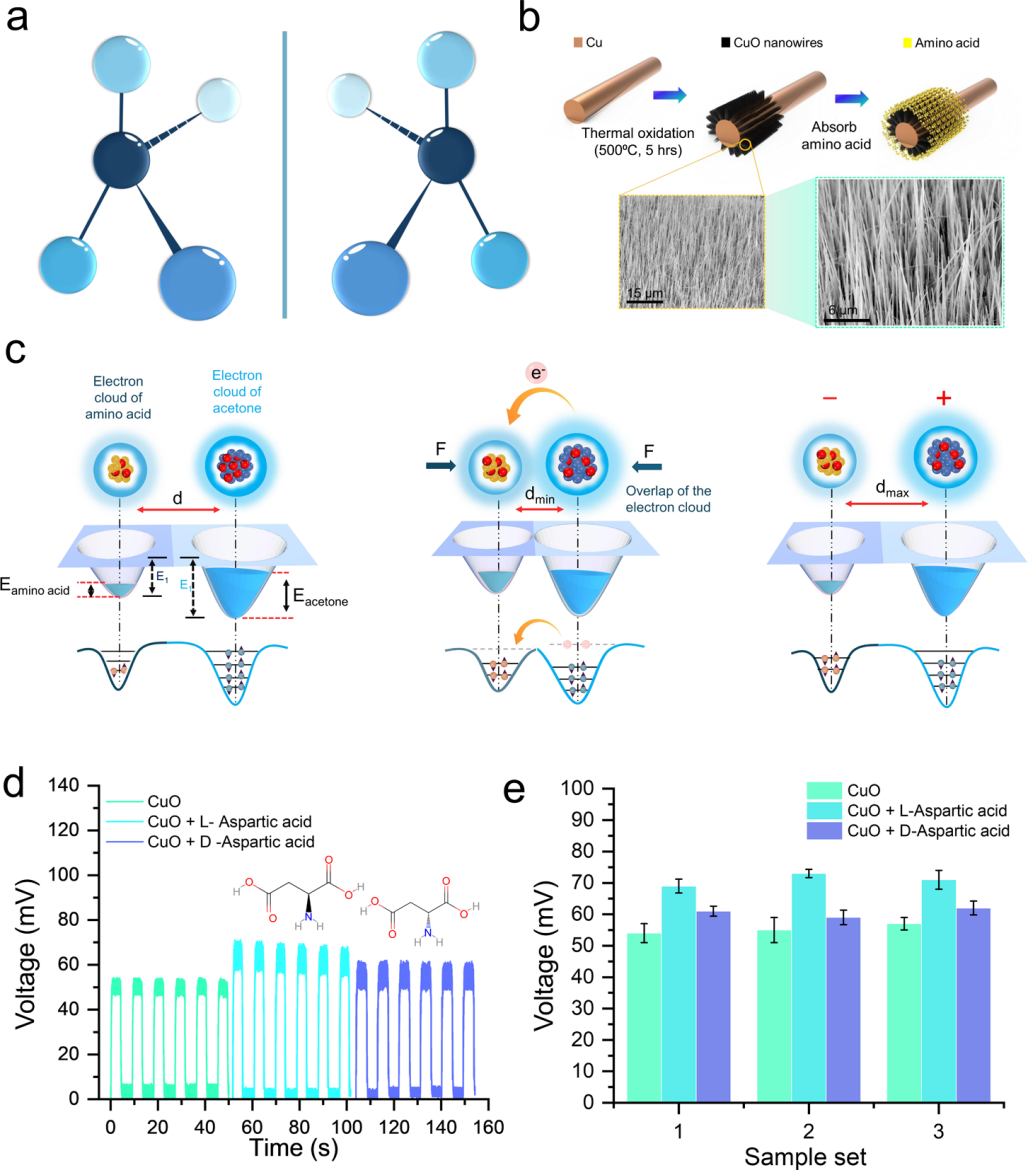
Stereospecific contact electrification for amino acid chirality recognition. (a) Schematic illustrations of l- and d-chiral amino acid molecular structures showing their mirror-image configurations. (b) Fabrication process of CuO nanowire growth, followed by amino acid absorption. SEM images show the resulting nanowire morphology. (c) Mechanism of chiral discrimination through contact electrification: electron cloud interactions between amino acids and acetone molecules during contact-separation cycles, showing energy profiles and electron transfer processes in three stages. (d) Voltage output over time comparing pristine CuO nanowires with l-aspartic acid and d-aspartic acid coated surfaces. (e) Statistical comparison of output voltages across three sample sets, demonstrating consistent differences between pristine CuO and l-aspartic acid-coated surfaces.

### Surface Chemistry and Morphological Characterization of Chiral Interfaces on CuO Nanowires

The schematic illustration of the amino acid-coated CuO nanowire device demonstrates the systematic approach for surface modification and subsequent chiral recognition capabilities (Figure 2a). The X-ray photoelectron spectroscopy survey spectra in Figure 2b,c reveal the elemental composition between Cu and CuO samples. Figure 2b shows the characteristic peaks for metallic copper, with small Cu 2p_3/2_ and Cu 2p_1/2_ peaks, along with the satellite peaks and other elemental signatures, including oxygen (O 1s) and carbon (C 1s) peaks.^36,37^ In contrast, Figure 2c displays the XPS spectrum of CuO nanowires, showing the characteristic copper oxide peaks with distinct binding energy shifts compared to metallic copper, confirming successful oxidation to the CuO phase.^38^ High-resolution XPS analysis in Figure 2d shows the Cu 2p spectrum for metallic copper, with the prominent characteristic Cu 2p_3/2_ peak at approximately 932.5 eV and low-intensity satellite peaks are indicative of the metallic state.^39,40^ Figure 2e presents the Cu 2p spectrum for CuO, displaying the shifted binding energies and prominent satellite peaks characteristic of Cu^2+^ oxidation state, confirming the formation of copper oxide nanowires.^41^ KPFM surface potential maps (Figure 2f,g) demonstrate the surface electronic properties of Cu and CuO surfaces. The copper surface (Figure 2f) exhibits a relatively uniform surface potential of 15 mV across the scanned area, while the CuO surface (Figure 2g) shows a different surface potential distribution of −139 mV, reflecting the increased work function upon oxidation.^42,43^ Furthermore, the SEM images (Figure 2h–k) reveal the surface morphological changes upon amino acid coating. These images confirm successful amino acid adsorption while maintaining the underlying nanowire structure, with morphological differences visible among various amino acid modifications. Moreover, XPS analysis of amino acid-coated surfaces (Figure 2l–m) provides crucial insights into the successful coating of the amino acid on the metal-oxide surface. The observed differences in XPS spectra between enantiomeric pairs demonstrate that l- and d-amino acids form coatings with different molecular orientations and packing arrangements, resulting in measurably different surface chemical environments. The high-resolution XPS peak analysis of the CuO surface after modification by l-/d-arginine and cysteine is represented in Figures S4–S7. Besides, a detailed analysis represented in the Supporting Information S3 confirms the successful coating of the amino acids on CuO nanowires. The chirality-dependent electronic properties of the molecular coating provide the fundamental basis for the differential electrical responses observed in the triboelectric measurements, suggesting that the detection mechanism relies on genuine molecular recognition events rather than nonspecific interactions.

**Figure 2:**
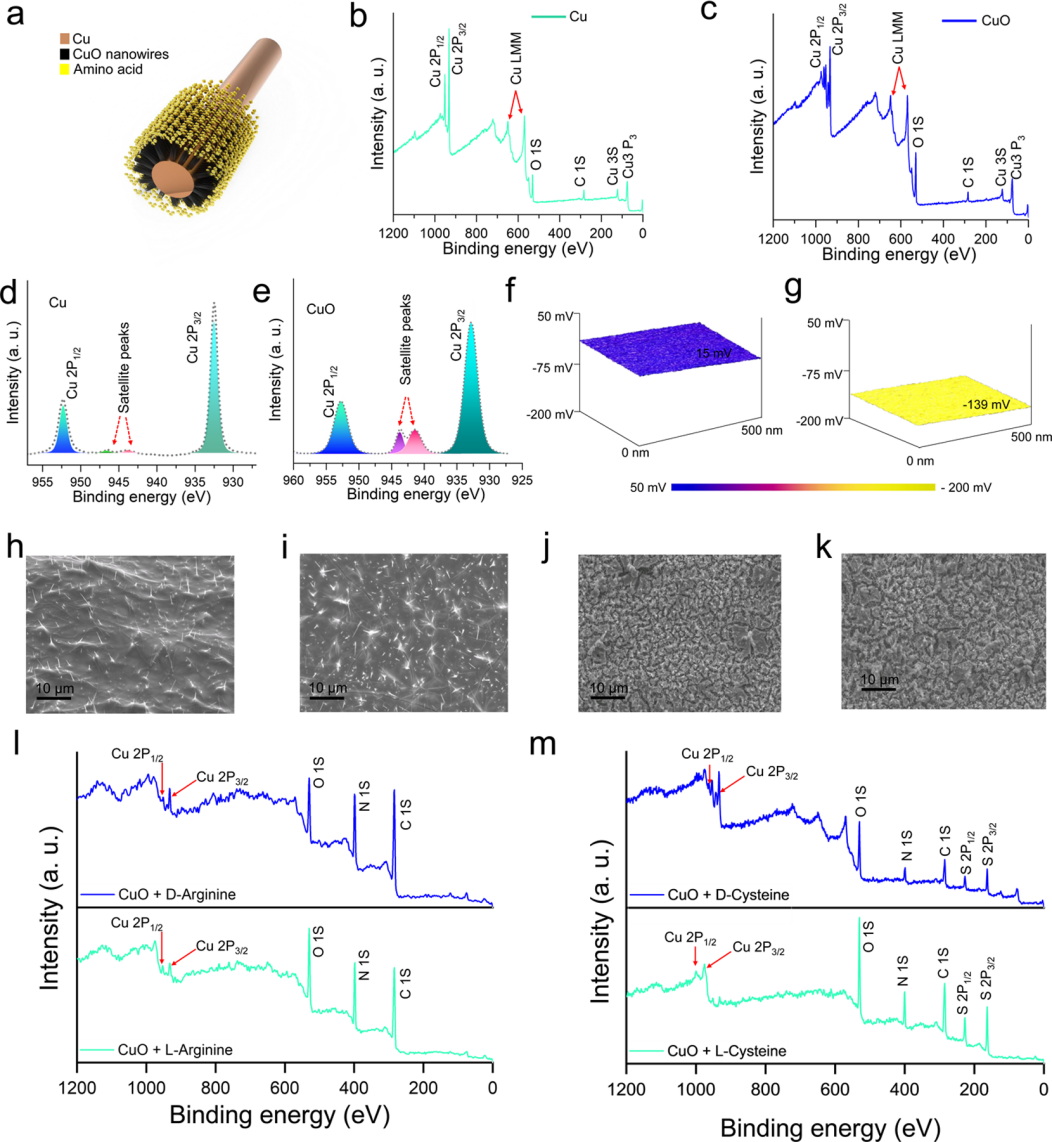
Structural and electronic characterization of chiral CuO interfaces. (a) Schematic of the amino acid-coated CuO nanowire device. (b,c) XPS survey spectra of Cu and CuO samples. (d,e) High-resolution XPS spectra of Cu 2p regions for Cu and CuO samples. (f,g) (KPFM) surface potential maps of Cu and CuO surfaces. (h–k) SEM images showing surface morphology of amino acid modified CuO nano wires; (h) d-arginine, (i) l-arginine, (j) d-cysteine, and (k) l-cysteine modified sensing surface. (l–m) Comparative XPS spectra of CuO surfaces after absorption of d-/l-Arginine and d-/l-cysteine, revealing differences in surface chemical compositions based on chirality.

### Electronic Structure Origins of Enantioselective Triboelectric Signals

The presented circular dichroism (CD) spectra of l-/d-arginine and l-/d-cysteine enantiomeric pairs at 2.5 mM concentration exhibit characteristic mirror-image patterns between enantiomers (Figure 3a,b). The CD spectra, with l-arginine showing positive Cotton effects around 220–240 nm while d-arginine displays corresponding negative signals. Similarly, l-cysteine and d-cysteine demonstrate opposing CD signatures, confirming the preservation of molecular chirality in solution and validating the enantiomeric purity of the amino acid samples used in the detection platform. Figure 3c presents UPS measurements revealing distinct work function values for CuO surfaces coated with different chiral amino acids. These work function differences provide the fundamental electronic basis for the observed triboelectric sensing capabilities, where different energy level alignments between enantiomeric amino acid-coated surfaces result in varying charge transfer efficiencies. The molecular orbital energy level diagrams (Figure 3d–f) present DFT calculations comparing the HOMO–LUMO energy levels of d-/l-arginine, d-/l-cysteine, and acetone systems. The calculated energy values demonstrate measurable differences between enantiomeric pairs, with HOMO and LUMO energy levels varying between l- and d-forms of the same amino acid. More importantly, the HOMO values are closely related to the ionization potential (IP) and the work function of these molecules.^44,45^ Therefore, the computational results support the experimental observations of chirality-dependent electronic properties and provide theoretical insight into the molecular basis for enantiomer discrimination. Figure 3g,h displays the output voltage measurements from CuO surfaces coated with l-/d-enantiomers of arginine and cysteine, respectively. The voltage outputs show clear discrimination between l- and d-forms of arginine, with consistent peak amplitudes around 127 mV, and 150 mV (Figure 3g), and for three consecutive sets of both enantiomer-based devices, confirms the same trend of output (Figure S8). Besides, these sensors achieve a response time of 500 ms (Figure S9). Similarly, cysteine enantiomers (Figure 3h) exhibit distinguishable electrical signatures, with voltage amplitudes of 82 mV for l-cysteine and 72 mV for d-cysteine. Furthermore, the three sets of devices for these enantiomers confirm the similar output trend that enables reliable molecule-specific chiral recognition (Figure S10).

**Figure 3:**
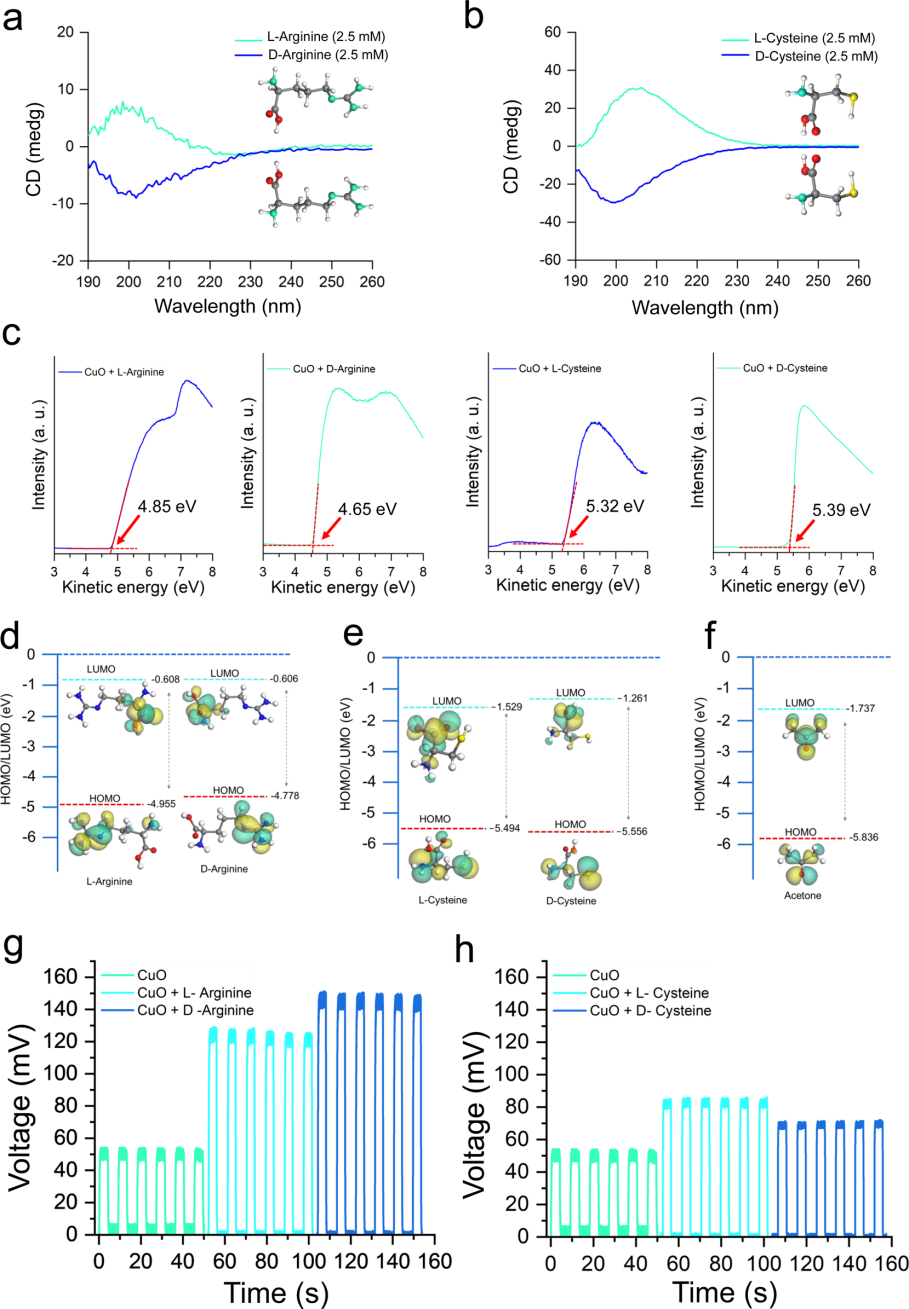
Electronic structure basis of amino acid-specific enantioselective triboelectric outputs. (a,b) Circular dichroism (CD) spectra of l-/d-arginine and l-/d-cysteine (2.5 mM). (c) UPS measurements work-function values for CuO surfaces with different chiral amino acid coatings. (d–f) Molecular orbital energy level diagrams comparing HOMO–LUMO gaps of d-/l-Arginine, d-/l-Cysteine, and acetone, with calculated energy values using DFT calculations. (g,h) The output voltage measurements showing distinct electrical signatures from CuO surfaces coated with l-/d-enantiomers of Arginine and Cysteine.

### Nanoscale Mapping of Chirality-Dependent Surface Potentials and Work Functions

Further investigation of the enantioselective charge transfer mechanism at the nanoscale, KPFM measurements were performed. KPFM scanning was performed on amino acid-coated CuO surfaces before and after 500 cycles (i.e., near about 75 min) of CE with acetone. The experimental configuration for surface potential mapping using KPFM is illustrated in Figure 4a, while the corresponding energy band diagrams (Figure 4b) depict the principle of work function equilibration and potential difference. Three-dimensional surface potential maps obtained from KPFM reveal distinct chirality-dependent electronic behaviors. Before contact, CuO surfaces modified with l-arginine exhibit an average surface potential of approximately 393 mV, which increases to 418 mV after contact (Figure 4c). Similarly, d-arginine-coated surfaces display a higher initial surface potential than l-arginine (455 mV), shifting to 488 mV postcontact (Figure 4d). These results suggest that d-arginine promotes greater charge transfer at the interface than its l-enantiomers. Histogram analyses of the surface potential distributions (Figure 4e) confirm this trend, revealing statistically significant shifts in surface potential upon contact with acetone, with the magnitude of change being enantiomer dependent. A similar pattern was observed for cysteine-coated CuO surfaces. However, here d-cysteine-modified surfaces demonstrated a precontact potential of −99 mV, which increased to −89 mV after contact (Figure 4f), while l-cysteine surfaces potential (mapping) shifted from −55 mV to −41 mV (Figure 4g). Surface potential histograms (Figure 4h) again revealed molecule-specific chirality-dependent shifts, indicating differential surface charging behavior induced by the stereochemical configuration of the functional groups. To visualize and compare the enantioselective surface charging among samples, polar radar plots were generated for both arginine (Figure 4i) and cysteine (Figure 4k). These plots capture the average surface potential variations before and after contact, demonstrating unique directional trends for each enantiomer pair. Furthermore, work function values calculated from the KPFM data are summarized in Figure 4j,l. Herein, the work function decreased after contact for all samples, but the degree of change differs between l- and d-enantiomers. Moreover, the cysteine pair shows smaller differences than the arginine pair before and after contact. The Supporting Information S4 provides a comprehensive explanation and describes the importance of the surface potential shift-based nanoscale mechanism for enantiomer discrimination, using KPFM. This highlights the influence of side chain functionality and the energy label gaps on chiral charge transfer efficiency between the contact materials.

**Figure 4:**
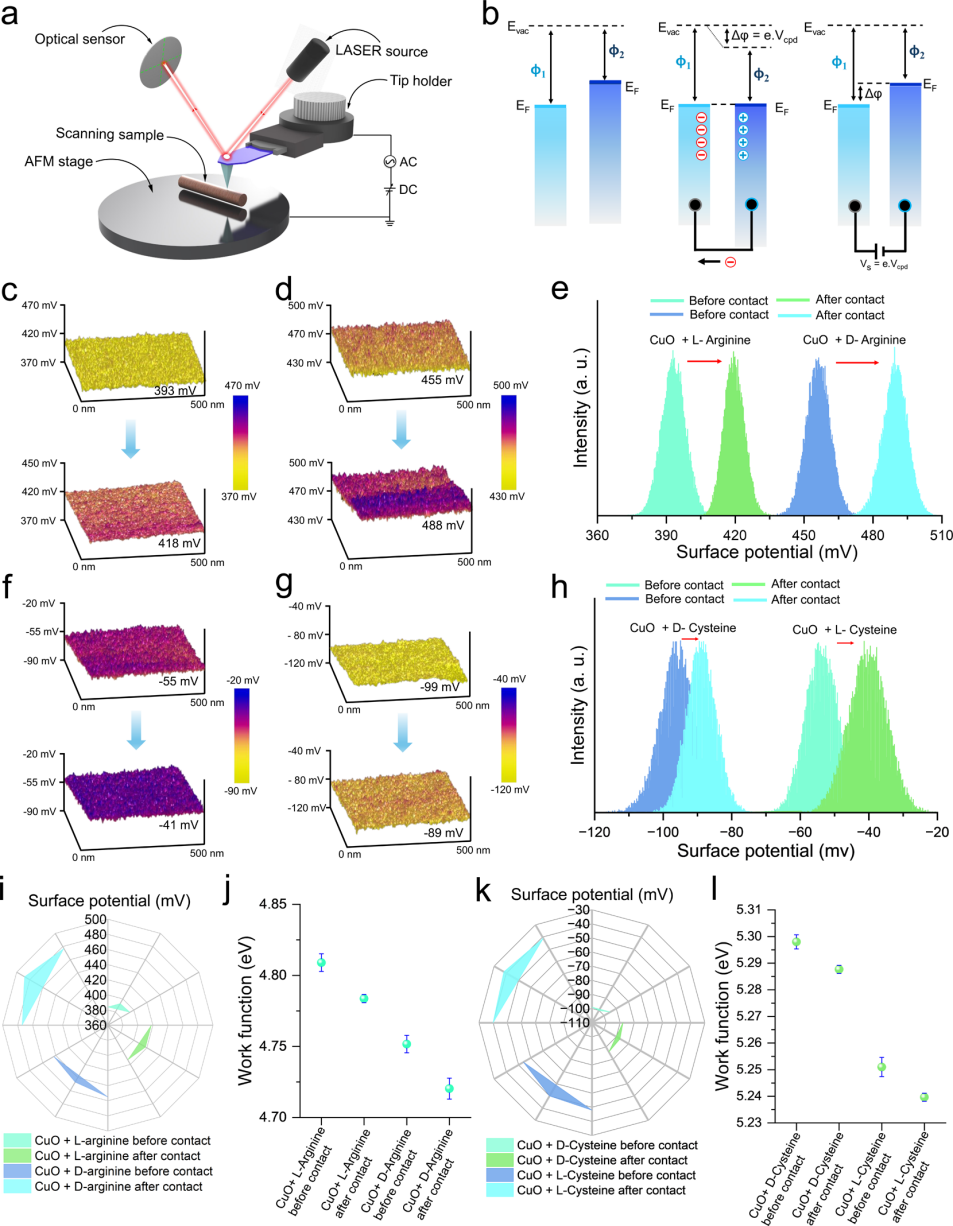
Nanoscale origin of stereospecific charge transfer revealed by KPFM. (a) Schematic illustration of the KPFM setup. (b) Energy band diagrams showing work function measurement process using KPFM. (c) The surface potential mapping of l-arginine and (d) d-arginine coated CuO surfaces before and after the CE process. (e) Surface potential distributions for CuO with l-/d-arginine before and after contact electrification. (f) Surface potential mapping of l-cysteine, (g) d-cysteine coated surfaces before and after contact. (h) Surface potential distributions for CuO with l-/d-cysteine before and after contact, demonstrating characteristic shifts based on chirality. (i) Polar plot representation of surface potential data for arginine enantiomers under different contact conditions. (j) Quantitative work function analysis of l-/d-arginine-coated surfaces. (k) Radial surface potential mapping for cysteine enantiomer systems. (l) Quantitative work function of l- and d-cysteine coated surfaces.

Besides, based on the fundamental relationship between surface charge density and surface potential, surface charge density (σ) can be calculated using eq 1 in the case of solid–liquid CE. In this work, σ is calculated using KPFM measurements in the air medium, after the CE process.

1 σ=ε0×ΔΦd

Herein, ε_0_ is the permittivity of free space (8.854 × 10^–12^ F·m^–1^), and ΔΦ represents the surface potential change of the amino acid-modified CuO surfaces after CE. The *d* is the effective KPFM probe-to-surface separation distance during surface potential mapping.^42,43,46–53^

Table 1 represents the surface potential variations and corresponding surface charge densities for the chiral amino acid modified sensor surfaces, before and after contact electrification. Moreover, detailed surface charge calculation is mentioned in Supporting Information S5. The surface charge densities were calculated using the equation σ = ε_0_ × ΔΦ/*d* with *d* = 100 nm. The 100 nm value was selected based on typical lift heights used in KPFM measurements. This distance is consistent with values reported in similar triboelectrification-based surface charge studies.^54–58^ The absolute values depend on this distance parameter used in the calculation. However, the relative differences between enantiomers are independent of d and demonstrate clear stereospecific charge transfer. d-arginine shows approximately 32% higher charge density than l-arginine under identical measurement conditions. In contrast, d-cysteine shows approximately 28% lower charge density than l-cysteine under the same conditions. The relative surface charge densities extracted from KPFM measurements provide quantitative evidence for stereochemical effects. These results demonstrate that elusive stereochemical differences in molecular geometry directly modulate charge transfer efficiency at nanoscale. Thus, it is confirmed that the CE mechanism involves discrete charge transfer events at molecular contact points, governed by quantum mechanical tunneling and orbital overlap dynamics. Nanoscale surface roughness and molecular heterogeneity create multiple contact asperities that contribute to overall charge transfer through localized electric field enhancement and chirality-specific molecular recognition effects. Additionally, the devised sensors exhibit a very stable output performance for 1 h through continuous contact separation cycles and prove reusability until the fifth day if the sensor is stored properly in a vacuum container (Figure S11). Further, quantitative sensor characterization across concentration ranges of 0.005–5 mM reveals excellent analytical performance for d-arginine detection (Figure S12). The calibration curve demonstrates strong linearity with a limit of detection (LOD) of approximately 1.7 μM (Figure S12). The sensor maintains consistent performance across 3 orders of magnitude, enabling quantitative analysis without sample dilution. Besides, Figure S13 represents the selectivity test using a racemic mixture of l-/d-arginine. This demonstrates the excellent selectivity of the proposed sensor toward side-chain specific detection capability. Critically, these metrics are achieved while preserving the unique operational advantages of the sensors. The advantages are a 500 ms response time enabling real-time monitoring, completely label-free detection, and simple operation without complex sample preparation—features particularly valuable for high-throughput pharmaceutical quality control and point-of-care applications.

**Table 1:** Calculated Surface Charge Density Values

amino acid	surface potential before contact-electrification (mV)	surface potential after contact-electrification (mV)	Δ*V* (mV)	surface charge density (μC·m^–2^)
l-Arginine	393	418	+25	+22.1
d-Arginine	455	488	+33	+29.2
l-Cysteine	–55	–41	+14	+12.4
d-Cysteine	–99	–89	+10	+08.9

### Extending the Recognition Mechanism to Amino Acids with Polar Uncharged Side Chains

The proposed chiral recognition technique is validated through the successful detection of threonine enantiomers. Herein, these homochiral molecules were investigated as model compounds containing polar-uncharged side chains. The XPS survey spectra confirmed successful surface coating of CuO substrates with both l-threonine and d-threonine, as evidenced by the appearance of characteristic photoelectron peaks corresponding to Cu 2p, O 1s, N 1s, and C 1s binding energies (Figure 5a,b). Further, the high-resolution XPS analysis of CuO modified with l-/d-threonine are depicted in the Figures S14–S15, with comprehensive analysis in Supporting Information S3. KPFM surface potential mapping revealed distinct chirality-dependent electronic responses following CE with acetone solvent. d-threonine-modified surfaces exhibited an initial surface potential of approximately 50 mV, which increased to 75 mV after CE. Besides, l-threonine coated surfaces demonstrated a significantly lower baseline potential of ∼1 mV that elevated to 15 mV post-CE (Figure 5c,d). Statistical analysis of surface potential distribution histograms confirmed these enantiomer-specific shifts, with both chirality demonstrating positive potential changes of markedly different magnitudes upon acetone interaction (Figure 5e). UPS measurements explained the underlying electronic structure differences, revealing distinct work function values of 5.17 eV for l-threonine and 5.00 eV for d-threonine modified surfaces (Figure 5f,g). Density functional theory calculations of molecular orbital energies provided further mechanistic insight, showing differentiated HOMO–LUMO gaps. For l-threonine, the LUMO energy level at −2.110 eV and HOMO level at −5.400 eV. Besides, for d-threonine LUMO energy level at −1.542 eV, and HOMO levels at −5.248 eV respectively (Figure 5h). These electronic structure variations directly correlate with the observed chiral selectivity in triboelectric charge transfer processes. Moreover, the measured voltage output demonstrated reproducible and distinct electrical signatures. Here, l-threonine consistently generates a lower voltage responses (∼83 mV) compared to d-threonine (∼99 mV) (Figure 5i). Further, statistical analysis across three independent sample preparations confirmed the reproducibility and statistical significance of these chirality-dependent performance differences (Figure 5j). Thereby validating the proposed molecular-level mechanism for molecule-specific enantioselective triboelectric charge transfer in amino acids possessing polar uncharged functional groups.

**Figure 5:**
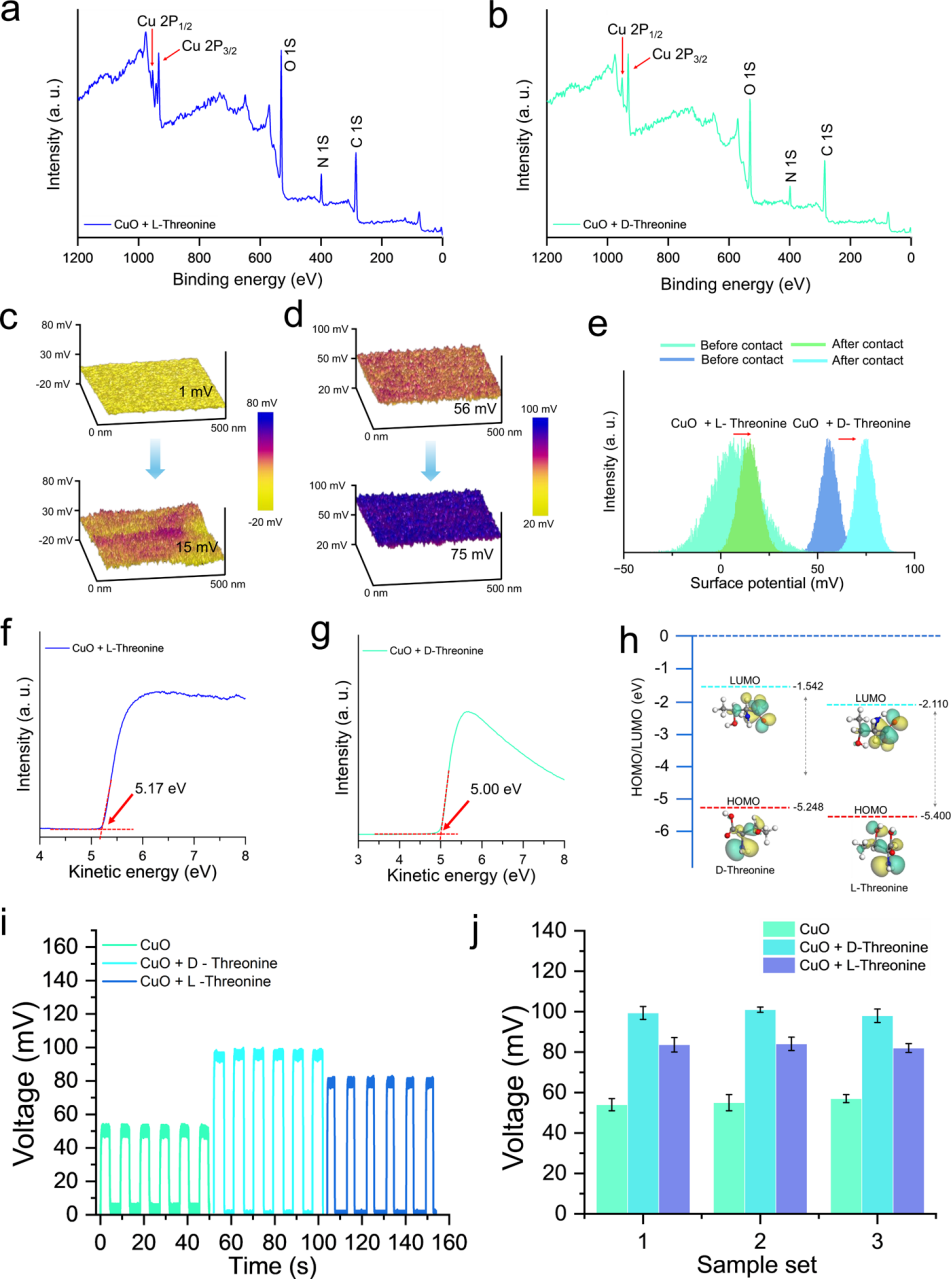
Extending the recognition mechanism to polar uncharged amino acids. (a,b) XPS survey spectra of CuO coated with l-threonine and d-threonine. (c) KPFM surface potential maps before and after contact for l-threonine and (d) d-threonine coated CuO surfaces. (e) Surface potential distributions of CuO with l-/d-threonine before and after contact electrification is represented. (f,g) The UPS measurements showing difference in work function for l-/d-threonine on CuO. (h) Molecular orbital energy diagram comparing HOMO–LUMO gaps of l-/d-Threonine with calculated energy values. (i) The depicted voltage output measurements showing distinct electrical signatures for amino acid enantiomers. (j) Statistical comparison of output voltages across three sample sets demonstrating consistent performance differences.

### Orthogonal Validation via Enantioselective Contact-Electrocatalytic Dye Degradation

To further probe the variation of triboelectric charge transfer capabilities of homochiral amino acids, a contact-electrocatalytic experiment was conducted for visual confirmation. In this experiment, a polytetrafluoroethylene film was utilized as the counter material with amino acids for solid–solid CE process (Figure 6a). The mechanism relies on contact electrification-induced charge transfer that modulates electron distribution between surfaces, thus subsequently affecting catalytic activity toward methyl orange degradation (Figure 6b,c). In this experiment, methyl orange solution was placed on the contact electrified PTFE surface as a droplet, after CE with different amino acid enantiomers. Then, after 5 min, the color change of the solution was analyzed using digital photography and optical measurements. This approach enables colorimetric detection of amino acid stereoisomers through differential catalytic responses through the methyl orange degradation. UV–vis absorption spectra revealed distinct enantiomeric discrimination capabilities across all tested amino acid pairs, with l- and d-cysteine showing characteristic spectral differences of methyl orange at 464 nm (Figure 6d). Furthermore, l- and d-threonine exhibited distinguishable absorption profiles of methyl orange (Figure 6e), and l- and d-arginine demonstrated clear enantioselective responses (Figure 6f). Besides, photographic documentation of the colorimetric responses of the dye degradation after CE experiments provided visual confirmation of the discrimination mechanism. Control experiments without CE showed no color change (Figure 6g), while slight color differences can be observed for l- and d-cysteine (Figure 6h). In case of l- and d-threonine (Figure 6i), a visible degradation of methyl orange is detected. Most prominent distinct color variations of methyl orange were exhibited by the l- and d-arginine (Figure 6j) pair, thus validating the enantiomer-selective contact electrification-dependent nature of the catalytic process. Video S1 represents a real-time contact electrocatalysis-based color change experiment between PTFE and a d-cysteine coated surface for better understanding along with Figure 6k. The video showcases the visible color change for the amino acid having the smallest surface charging among the examined amino acids. l-cysteine showed ∼20% degradation compared to ∼18.3% for d-cysteine, l-threonine achieved ∼36.7% versus ∼38.3% for d-threonine, and l-arginine reached ∼45%, while d-arginine showed ∼51.7% degradation efficiency (Figure 6l–n), as mentioned in Supporting Information Table S2. To summarize the results, three-dimensional radar plots provided comprehensive discrimination analysis across multiple parameters, including work function, surface potential, and catalytic activity, which clearly differentiated l- and d-arginine (Figure 6o), l- and d-cysteine (Figure 6p), and l- and d-threonine (Figure 6q) through their distinct multiparametric signatures. Thus, using the triboelectric approach provides a contemporary platform for rapid, label-free discrimination of amino acid enantiomers based on their intrinsic properties.

**Figure 6:**
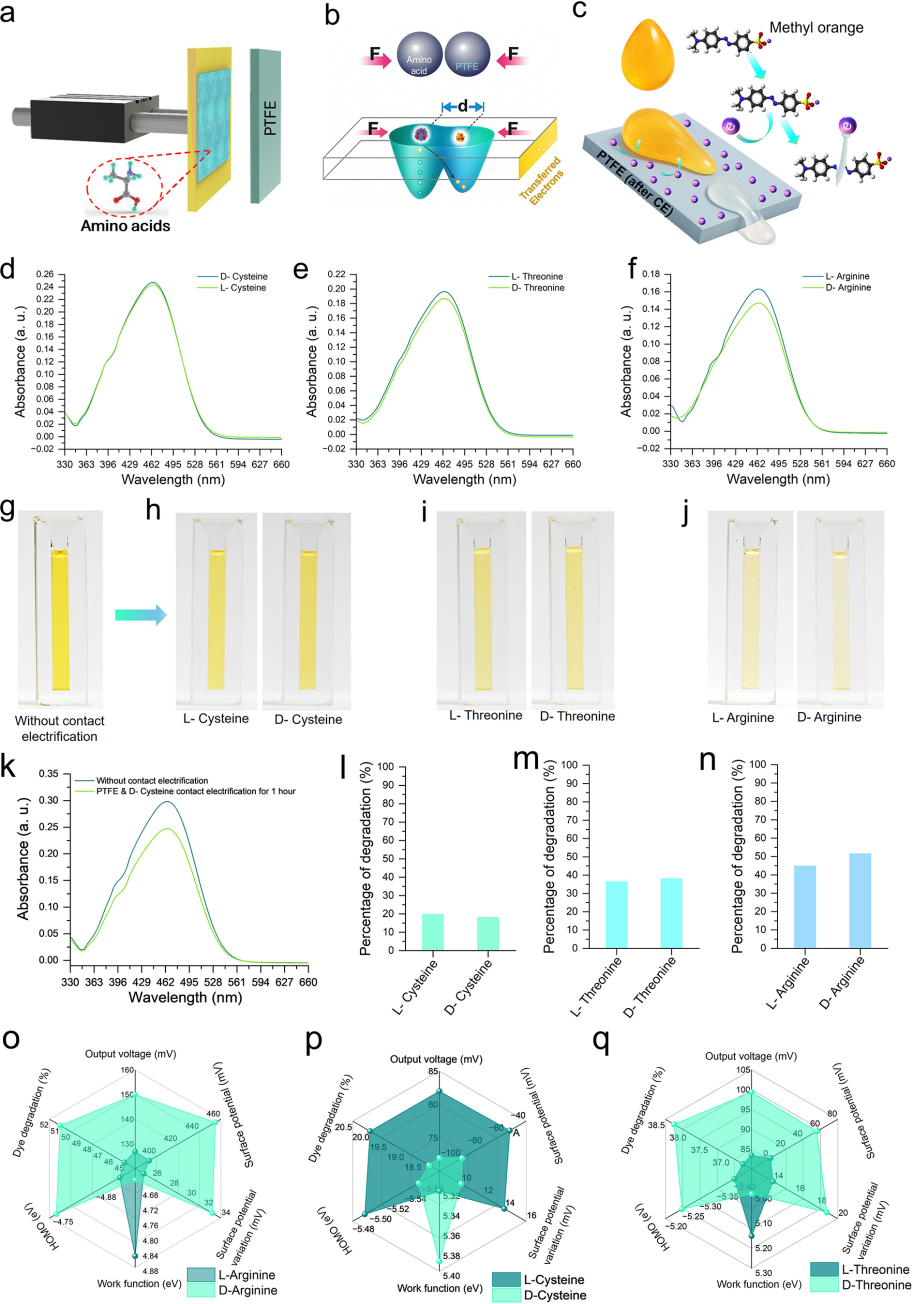
Orthogonal colorimetric validation of chiral recognition via contact-electrocatalysis. (a) Schematic illustration of the experimental setup employing PTFE counter material for amino acid contact electrification. (b) Mechanism of contact electrification-based charge transfer showing electron distribution between surfaces. (c) Catalytic degradation of methyl orange on the contact-electrified PTFE surface serving as a colorimetric indicator for enantioselective detection of amino acid stereoisomers. (d–f) UV–vis absorption spectra demonstrating enantiomeric discrimination for: (d) l-/d-cysteine, (e) l-/d-threonine, and (f) l-/d-arginine enantiomers. (g–j) photographic records of colorimetric responses during CE experiments: (g) control without contact electrification, (h) l-/d-cysteine differentiation, (i) l-/d-threonine differentiation, and (j) l-/d-arginine differentiation showing distinct color changes. (k–n) Comprehensive discrimination analysis showing: (k) UV–vis spectra comparison, and (l,n) percentage of degradation for l-/d-cysteine, l-/d-threonine, and l-/d-arginine, respectively. (o–q) Three-dimensional radar plots showing comprehensive discrimination analysis across multiple parameters for (o) l-/d-arginine, (p) l-/d-cysteine, and (q) l-/d-threonine comparison.

## Experimental Section

### Chemicals

All amino acids, including l-arginine (98% purity), d-arginine (98% purity), l-cysteine (97% purity), d-cysteine (97% purity), l-threonine (98% purity), d-threonine (98% purity), l-aspartic acid (99% purity), and d-aspartic acid (99% purity), were purchased from Sigma-Aldrich and used without further purification. Copper wire (99.9% purity, 1 mm diameter) was obtained from Alfa Aesar. Hydrochloric acid (HCl, 37% analytical grade), acetone (99.5% HPLC grade), methyl orange (85% dye content), and PTFE sheets were procured from Merck. Aluminum tape was sourced from 3 M Corporation. Deionized water (18.2 MΩ·cm resistivity) was obtained from a Millipore Direct-Q water purification system and used throughout all experiments. All chemicals were stored under appropriate conditions according to manufacturer specifications and used within their recommended shelf life to ensure experimental reproducibility and data reliability.

### Characterization

Surface chemical composition and elemental analysis were performed using XPS on an ESCALAB 250Xi spectrometer (Thermo Fisher Scientific) equipped with a PHI VersaProbe II system. The high-resolution core-level spectra were deconvoluted using CASA XPS software to determine binding energies. Electronic structure characterization was conducted through UPS measurements using an ESCALAB 250Xi spectrometer with HeI excitation source (21.2 eV photon energy) under ultrahigh vacuum conditions to determine work function values and valence band positions. Morphological analysis of the CuO nanowires was carried out using field emission scanning electron microscopy (FE-SEM) with JEOL JSM-7600F and Hitachi S-4800 instruments operated at 10 kV acceleration voltage. Prior to imaging, samples were thoroughly dried, mounted on conductive substrates, and sputter-coated with platinum to enhance conductivity and image quality. Surface potential mapping and work function measurements were performed using amplitude-modulated BRUKER ICON Kelvin probe force microscopy (AM-KPFM) equipped with ScanAsyst mode to investigate the electronic properties and charge distribution across the amino acid coated surfaces. Valence band X-ray photoelectron spectroscopy (VB-XPS) analysis was conducted using the ESCALAB 250XI spectrometer with Al Kα X-ray source (1486.6 eV) to probe the electronic band structure of the materials. Chemical bonding and functional group identification were analyzed through FTIR spectroscopy using a PerkinElmer FTIR spectrometer (model C123672) equipped with an attenuated total reflection (ATR) accessory for direct sample analysis. The optical characterization, including fluorescence intensity measurements, was performed using a HITACHI F-7000 fluorescence spectrophotometer, while UV–visible absorption spectra were recorded on a JASCO V-670 spectrophotometer to monitor colorimetric changes during catalytic degradation experiments.

### Device Fabrication

CuO nanowire-based triboelectric sensors were fabricated through a controlled thermal oxidation process followed by chiral amino acid coating. Copper wire segments (3 cm length) were straightened and marked at 1 cm to define the active sensing area, then cleaned with deionized water, acetone, and ethanol before drying at 60 °C for 30 min. The 2 cm nonactive portion was protected with aluminum tape to prevent unwanted oxidation. The exposed 1 cm copper surface underwent acid etching using 1 M HCl for 10 min to remove native oxide layers, followed by thorough rinsing and drying at 60 °C. CuO nanowires were synthesized via thermal oxidation in a muffle furnace with a controlled heating profile at 2.5 °C/min ramp rate to 500 °C, maintained at 500 °C for 5 h under ambient atmosphere, then naturally cooled to room temperature. Amino acid coating was achieved through dip-coating the CuO nanowire section in 2.5 mM aqueous solutions of respective l- or d-amino acid enantiomers at 37 °C for 2 h under gentle agitation, followed by drying at 37 °C for 30 min. Further, to check the contact electrocatalytic activity, the solid–solid TENG was operated under a vertical contact separation configuration in which the amino acid layer was coated on the glass, and the other contact surface was a PTFE film adhered on the PMMA surface. A programmable linear motor was employed to perform the contact separation motion. Herein, the linear motor operates with a force is 1 N, at a frequency of 2 Hz. The separation distance is 1 cm, the separation speed is 0.0256 m sec–1, and the mechanical waveform for the linear motor is a sine wave, which involves the back-and-forth movement of the TENG. Moreover, the contact area of the TENG is (3 × 2) cm^2^.

### Electrical Characterization Setup

The triboelectric output performance of the chiral amino acid-coated CuO nanowire sensors was evaluated using a solid–liquid CE system. The experimental setup consisted of amino acid-modified CuO nanowire surfaces as the solid triboelectric layer and acetone as the contact solvent to generate chirality-dependent charge transfer responses. A precision vertical dip-coating apparatus provided controlled and reproducible contact-separation cycles between the coated nanowire electrode and the liquid medium, ensuring consistent mechanical parameters for reliable triboelectric signal generation. The frequency of the dip-coating apparatus is fixed at 0.25 Hz, and the separation distance is kept at 1 cm. The complete measurement system is illustrated in Figure S16, showing the integration of all components for systematic electrical characterization. Operating under single-electrode configuration, the sensing performance was quantified by monitoring changes in output voltage corresponding to different amino acid enantiomers during CE cycles. The generated alternating current (AC) voltage signals were converted to direct current (DC) signals using a full-wave bridge rectifier circuit composed of four silicon diodes to facilitate stable signal processing and data acquisition. Signal recording was accomplished using a portable Wi-Fi enabled data logger (Elite System) with real-time wireless data transmission capabilities for continuous monitoring and analysis. Ceramic capacitors (6.6 nF) were incorporated into the signal conditioning circuit to filter ambient electrical noise and stabilize the measurement signals, ensuring high signal-to-noise ratio for accurate enantiomer discrimination. The circuit diagram for the signal detection is depicted in Figure S17. The environmental parameters were maintained throughout all measurements, with relative humidity kept within a 40 ± 5% range and temperature stabilized at 25 ± 0.5 °C, as previous studies have demonstrated that humidity and temperature variations can significantly affect triboelectric output and surface charging due to moisture-induced charge dissipation pathways. The entire measurement setup was housed within a metallic enclosure functioning as a Faraday cage to eliminate electromagnetic interference from external sources and ensure measurement accuracy. During data acquisition, all high-voltage equipment and dehumidifiers in the black box were disconnected to minimize potential sources of electrical noise and interference, maintaining optimal conditions for sensitive triboelectric signal detection and chiral discrimination measurements.

### Determination of Work Function

The work function (WF) of the samples was determined by measuring the contact potential difference (CPD) using Kelvin probe force microscopy (KPFM). A single-crystal diamond-based conductive atomic force microscopy (AFM) tip (model AD-2.8-AS) served as the reference electrode for CPD measurements.^59,60^ The relationship between CPD and work function is described by

2 CPD=Φtip−Φsamplee

where “*e*” represents the elementary charge of an electron.

Prior to sample characterization, the tip’s work function (Φ_tip_) was calibrated using highly oriented pyrolytic graphite (HOPG) as a reference standard. HOPG has a well-established work function of Φ_HOPG_ = 4.6 eV.^15,16,61,62^ Following calibration, the tip work function was determined to be 5.2 eV based on a measured surface potential of 600 mV (Figure S18).

The sample work function was then calculated using

3 $$\Phi_{sample}=\Phi_{tip}-e\times {CPD}_{sample}$$

where CPD_sample_ represents the measured contact potential difference of the sample. This approach enables precise determination of the surface work function, which is critical for understanding the material’s surface potential and electrical properties.

To ensure measurement reproducibility, a minimum of three distinct amino acid coated CuO samples were characterized, with three different locations tested on each sample. All KPFM measurements were conducted under controlled environmental conditions. Humidity was maintained at 40 ± 1% using a dehumidifier, while temperature was controlled at 25 ± 0.5 °C through an air conditioning system (Figure S19). These controlled conditions were essential for obtaining reliable and reproducible work function measurements.

#### Work Function Calculation

The study used CPD measurements between a cantilever probe and sample surfaces to determine theirWFs. The relationship between contact potential difference and work function is given by

4 VCPD=Φtip−Φsamplee

The WF of the sample can be calculated from the tip WF and the measured CPD of the sample

5 $$\Phi_{sample}=\Phi_{tip}-e\left(V_{CPD}\right)$$

Using this approach, the surface potential values and corresponding work functions were determined for the amino acid samples:

l-arginine: surface potential = 393 mV = > WF = 5.2 eV - (393 × 10^–3^ eV) = 4.81 eV

d-arginine: surface potential = 455 mV = > WF = 5.2 eV - (455 × 10^–3^ eV) = 4.75 eV

l-cysteine: surface potential = −55 mV = > WF = 5.2 eV - (−55 × 10^–3^ eV) = 5.26 eV

d-cysteine: surface potential = −99 mV = > WF = 5.2 eV - (−99 × 10^–3^ eV) = 5.30 eV.

Primary equation for the surface charge calculation.

The fundamental relationship between surface charge density (σ) and surface potential (Φ) is given by

6 σ=ε0×ΔE=−ε0×(∂V∂n)

for triboelectric systems with surface potential measurements, this simplifies to

7 σ=ε0×ΔΦd

where: σ = surface charge density (C·m^–2^), ε_0_ = permittivity of free space (8.854 × 10^–12^ F·m^–1^), ΔΦ = change in surface potential before and after contact (V), and *d* = effective separation distance or probe-to-surface distance (m).

Alternative formulation for contact electrification.

For triboelectric contact electrification, the relationship can also be expressed as σ = C_dl_ × Φ_0_, where C_dl_ represents the double-layer capacitance.

8 $$\sigma =C_{dl}\times \Phi_{0}$$

where, C_dl_ represents the interfacial capacitance between the amino acid-modified surface and acetone. Charge transfer occurs through electron exchange mechanisms influenced by the distinct electron-donating/accepting properties and work function differences of chiral amino acid modifications. The observed chiral selectivity is consistant with l- and d-amino acid modifications create different surface electronic environments, producing distinct triboelectric responses with the same acetone solvent. Kelvin probe measurements after solvent evaporation capture these macroscopic surface potential changes, reflecting the cumulative nanoscale charge transfer events across the contact interface.

However, the KPFM measurements were carried out in the controlled ambient environment (i.e., air medium), and thus, the simpler capacitor model is more appropriate

9 σ=εeff×ΔΦd

where ε_eff_ is the effective permittivity of the medium (air ≈ ε_0_). Thus, in our case,

10 σ=ε0×Φafter−Φbefored

### Computational Setup

For the theoretical prediction of HOMO–LUMO energy levels and electronic properties of chiral amino acids, DFT calculations were performed using the DMol3 module. The generalized gradient approximation (GGA) with Perdew–Burke–Ernzerhof (PBE) functional was employed to describe the exchange-correlation interactions. Geometry optimization was conducted prior to electronic structure calculations using coarse quality settings for computational efficiency. The convergence criteria were set as follows: first energy convergence of 1.0 × 10^–4^ Ha, maximum force tolerance of 0.02 Ha/Å, and maximum displacement of 0.05 Å. The optimization was limited to a maximum of 55 iterations with a maximum step size of 0.3 Å. The TS method was utilized for DFT-D dispersion correction to account for weak intermolecular interactions. Spin-unrestricted calculations were performed, and molecular symmetry was employed where applicable to reduce computational cost.

All molecular structures were constructed as neutral species (charge = 0) and were fully optimized before electronic property calculations. The calculations were performed without periodic boundary conditions, treating the amino acid molecules as isolated systems. The HOMO–LUMO energy gaps and molecular orbital distributions were analyzed to understand the electronic properties and chirality effects of the amino acid systems. Moreover, for the simulation of the energy levels of the acetone, we used the solvent mode to obtain a more realistic result. All the atomic positions and coordinates of the chiral molecules, before and after the geometrical optimization steps, are mentioned in Supporting Information S6 for a better understanding of the computational outcomes.

### Description of Contact Electrocatalysis

When chiral amino acids and PTFE undergo CE for 1 h, electrons transfer from the amino acid (l-arginine → l-arginine^+^ + e^–^) to the highly electronegative PTFE surface (PTFE + e^–^ → PTFE^–^), leaving l-arginine positively charged and PTFE negatively charged with a high surface charge density of approximately 29.2 μC·m^–2^. Upon placing the methyl orange solution on the negatively charged PTFE surface (for 5 min), the stored electrons are transferred to dissolved oxygen to generate reactive oxygen species through the sequence: PTFE^–^ + O_2_ → PTFE + ^•^O_2_^–^ (superoxide radical), followed by ^•^O_2_^–^ + H^+^ → HO_2_^•^ → ^•^OH (hydroxyl radical). These highly reactive hydroxyl radicals then degrade the methyl orange molecules in an oxidative degradation process: C_14_H_14_N_3_NaO_3_S + ^•^OH → colorless intermediate products, ultimately leading to mineralization. The solution exhibits a colorimetric transition from yellow to colorless, or a diminution in yellow chroma, indicating a decrease in the concentration of the chromophore responsible for the yellow coloration the color change occurs because the reactive oxygen species destroy the chromophoric azo bond (–N=N–) and aromatic ring structures responsible for the characteristic orange color at ∼465 nm absorption, demonstrating that even though PTFE becomes negatively charged, it facilitates oxidative (not reductive) degradation of the dye by serving as an electron reservoir for generating oxidizing radicals rather than directly reducing the organic molecule.^46–53,63–66^

## Conclusions

This work provides a mechanistically grounded chemical sensing platform in which molecular chirality is directly transduced into macroscopic electronic signals through contact electrification. By coating CuO nanowires with enantiopure amino acids, we create chiral interfaces whose orbital energy alignment and surface work function are inherently dependent on side-chain geometry and binding configuration. Controlled contact–separation with acetone elicits enantioselective charge transfer events, producing distinct triboelectric outputs within milliseconds. Correlating triboelectric signals with Kelvin probe force microscopy, ultraviolet photoelectron spectroscopy, and density functional theory calculations reveals that chirality-dependent differences in nanoscale surface potential and electron cloud overlap influence the efficiency of interfacial charge transfer. Orthogonal supportive corelation via contact-electrocatalytic dye degradation further demonstrates that these electronic disparities manifest in measurable differences in chemical reactivity, linking molecule-specific stereochemistry to macroscopic catalytic outcomes. Beyond enabling rapid, label-free recognition of diverse amino acids—including charged, polar uncharged, and sulfur-containing structures—this approach reframes chiral sensing as an interfacial electron transfer problem rather than solely a structural recognition challenge. The integration of triboelectric transduction with stereospecific electronic structure control offers a versatile framework for detecting a wide range of chiral molecules in contexts spanning pharmaceutical quality control, asymmetric catalysis monitoring, and biomolecular diagnostics. By bridging molecular recognition chemistry with solid–liquid interfacial physics, this study expands the conceptual and methodological toolbox for enantioselective analysis, providing a route toward energy-autonomous, mechanistically transparent, and chemically generalizable chirality sensing.

## Supplementary Material





## Notes

Experimental details of triboelectric nanosensor fabrication, chiral amino acid coatings, and comprehensive characterization including SEM, KPFM, XPS, UPS, FTIR, CD spectroscopy, DFT calculations, contact electrification measurements, and contact-electrocatalytic validation experiments (PDF)
Video S1 represents a real-time contact electrocatalysis-based color change experiment between PTFE and a d-cysteine coated surface for better understanding along with Figure 6k (MP4)
