# Supplementary material for: Contact Electrification–Based Enantioselective Recognition of Chiral Amino Acids through Stereospecific Interfacial Electron Transfer
Source: J Am Chem Soc. 2026 Jan 12;148(3):3063–76. doi: 10.1021/jacs.5c15608 (PMC12856897; doi:10.1021/jacs.5c15608)
Supplement: Supplementary file 1 [file ja5c15608_si_001.pdf]

Contact Electrification–Based Enantioselective Recognition of Chiral Amino Acids through Stereospecific Interfacial Electron Transfer

Arnab Pal[†], Hakjeong Kim[‡], Shreerag Suresh[†], Po-Han Wei[†], Abdullah Mohamed Al-Kabbany[†], Du-khyun Choi[‡], and Zong-Hong Lin[†]*

[†]Department of Biomedical Engineering, National Taiwan University, Taipei 10617, Taiwan
[‡]School of Mechanical Engineering, College of Engineering, Sungkyunkwan University, Suwon 16419, South Korea

* Corresponding authors: zhlin@ntu.edu.tw

Supplementary Information Contents

a. Supplementary Notes

1. **Supplementary Note 1**: Working principles of contact electrification in chiral recognition
2. **Supplementary Note 2**: Physicochemical properties of contact liquid solvents: comparative analysis for chiral amino acid detection
3. **Supplementary Note 3**: XPS evidence of covalent chiral coating on CuO nanowires
4. **Supplementary Note 4**: KPFM mapping reveals nanoscale origins of enantioselective charge transfer
5. **Supplementary Note 5**: Surface charge density calculations
6. **Supplementary Note 6:** Optimized geometries from DFT calculations

b. Supplementary Tables

1. **Table S1**: Comparative analysis of chiral molecule detection techniques
2. **Table S2**: Methyl orange degradation percentages

c. Supplementary Figures

1. **Figure S1:** Working mechanism illustration of CuO-nanowire-based solid-liquid TENG
2. **Figure S2:** FTIR spectra analysis of CuO before and after chiral amino acid coating
3. **Figure S3:** SEM and EDS characterization of CuO nanowires modified with chiral amino acids
4. **Figure S4:** High-resolution XPS analysis of CuO modified with L-arginine
5. **Figure S5:** High-resolution XPS analysis of CuO modified with D-arginine
6. **Figure S6:** High-resolution XPS analysis of CuO modified with L-cysteine
7. **Figure S7:** High-resolution XPS analysis of CuO modified with D-cysteine
8. **Figure S8:** Statistical comparison of output voltages for L/D-arginine detection
9. **Figure S9:** Electrical response characteristics of chiral amino acid-modified CuO TENG
10. **Figure S10:** Statistical comparison of output voltages for L/D-cysteine detection
11. **Figure S11:** Long-term stability and reusability of D-Arginine modified sensor

12. **Figure S12:** Quantitative concentration-dependent sensor performance and analytical metrics
13. **Figure S13:** Selectivity test of the sensor using racemic mixtures of L- and D-arginine enantiomers
14. **Figure S14:** High-resolution XPS analysis of CuO modified with L-threonine
15. **Figure S15:** High-resolution XPS analysis of CuO modified with D-threonine
16. **Figure S16:** Digital image of solid-liquid contact electrification-based detection system
17. **Figure S17:** Circuit diagram of wireless data transmission and acquisition process
18. **Figure S18:** Contact potential difference calibration using HOPG reference standard
19. **Figure S19:** Experimental setup for characterizing the chiral amino acid-modified CuO TENG surface before and after contact electrification, under controlled environmental conditions using KPFM

d. References for supplementary Information

Supplementary Note 1.

Working principles of contact electrification in chiral recognition

Figure. S1 Illustrates the working mechanism of the CuO-nanowire-based solid-liquid triboelectric nanogenerator (TENG), which operates on the fundamental principles of contact electrification and electrostatic induction. The static surface charges are generated through contact electrification, while electrostatic induction drives electron flow under the potential difference created by applied mechanical force. In our experimental setup, the amino acid-coated CuO nanowires and acetone contact liquid are subjected to controlled mechanical force through a dip coater to produce periodic contact-separation movements, generating triboelectric outputs.

When the amino acid-coated CuO nanowire arrays are immersed in acetone (**Figure. S1i**), contact electrification occurs due to their different electron affinities according to the triboelectric series. Given that acetone is a volatile organic solvent and most amino acids are insoluble in acetone, this solvent choice ensures stable interfacial interactions. During contact, electrons transfer from the CuO nanowire surface to the acetone, causing the solid surface to acquire positive charges while the liquid surface becomes negatively charged. At full contact, the triboelectric charges on both surfaces are completely balanced, resulting in no electron flow through the external circuit.

As the amino acid-coated CuO surface is gradually withdrawn from the acetone (**Figure. S1ii-iii**), the equilibrium state is disrupted while the CuO surface retains its positive charges. To maintain electrical neutrality, electrons flow from the ground through the external circuit to the CuO electrode. The magnitude of this output current is proportional to the potential difference between the CuO surface and ground. This electron flow continues until the equilibrium is reached and the CuO surface becomes completely separated from the acetone.

When the CuO surface begins to re-immerse in the acetone (**Figure. S1iv**), an opposite potential difference is generated, driving electron flow from the CuO electrode back to the ground to restore electrical neutrality. This creates a current in the opposite direction through the external circuit. The process continues until full contact is re-established and the potential difference returns to zero, completing the cycle and restoring the original state (**Figure. S1i**).

The choice of acetone as the contact liquid is particularly advantageous because its volatile nature and the insolubility of amino acids in acetone ensure that the surface properties of the amino acid-coated CuO nanowires remain stable throughout the periodic contact-separation cycles. This perpetual contact-separation process between the acetone and amino acid-coated CuO electrode results in continuously varying output voltages, enabling effective sensing performance from mechanical motion.

The measured triboelectric voltage arises from stereospecific electron transfer occurring directly between chiral amino acid molecules and acetone during contact separation cycles. The amino acids function as active recognition elements participating in charge exchange rather than serving as passive surface coatings. DFT calculations reveal distinct HOMO LUMO gaps between enantiomers, while UPS measurements show different work functions, and KPFM analysis demonstrates chirality dependent surface charge densities. CuO nanowires provide the conductive scaffold, enabling the chiral amino acids to constitute the functional interface where stereospecific molecular recognition and electron transfer occur. While the present work establishes strong correlations between electronic structure parameters and triboelectric outputs, a fully predictive physical model incorporating electron tunneling rates, charge accumulation dynamics, and interfacial capacitance remains an important goal for future work. Such modeling requires detailed knowledge of transient interfacial contact geometry, barrier parameters during mechanical oscillation, and surface state distributions—parameters that are challenging to access experimentally in dynamic contact-separation systems. The correlations established here provide the experimental foundation for developing such quantitative frameworks, analogous to the trajectory of triboelectric nanogenerator research where phenomenological demonstrations preceded comprehensive theoretical models.

Supplementary Note 2.

Physicochemical properties of contact liquid solvents: comparative analysis for chiral amino acid detection

Table for Physicochemical Properties of Contact Liquid Solvents (at 20-25°C)

Solvent	Dielectric Constant (ε)	Boiling Point (°C)	Vapor Pressure (kPa)	Volatility Classification	Amino Acid Solubility	Reference
Hexane	2.02	69	17.6	High	Insoluble	1-14
Isopropyl Alcohol	~19	82.3	4.4	Medium	Low-Moderate	1-14
**Acetone**	**20.7**	**56**	**30**	**Very High**	**Insoluble**	1-14
Acetonitrile	36.6	81.6	~11	High	Low-Moderate	1-14
Ethanol	24.3	78.3	12.4	High	Moderate-High	1-14
Water	80.0	100	2.4	Low	Very High	1-14

The choice of acetone as the contact solvent was strategically justified by several critical factors essential for maintaining a stable chiral interface. As explicitly stated in Supplementary Note 1, acetone's volatile nature, combined with the insolubility of amino acids in this solvent, ensures that "the surface properties of the amino acid-coated CuO nanowires remain stable throughout the periodic contact-separation cycles." This prevents dissolution of the chiral functional layer, thereby maintaining the precise molecular architecture and orientation required for enantioselective recognition.(15,16) Additionally, acetone's moderate polarity (dielectric constant $\varepsilon \approx 20.7$) and its position in the triboelectric series relative to amino acid-coated CuO enable effective charge transfer during contact electrification, while its rapid evaporation prevents accumulation or contamination between measurement cycles.(17,18)

Alternative solvents would likely produce dramatically different results based on fundamental physicochemical considerations. Water, despite being an ideal solvent for most biological applications, would be highly problematic as it readily dissolves amino acids, leading to destruction of the chiral interface and yielding unstable, irreproducible signals.(19) High-polarity aprotic solvents such as acetonitrile might partially dissolve amino acids or significantly alter their binding orientations and conformations through competitive dipole-dipole interactions and potential hydrogen bonding with residual water, thereby disrupting the stereospecific surface architecture.(20,21) Conversely, non-polar solvents like hexane would generate substantially weaker charge transfer due to poor electronic coupling with the polar, zwitterionic amino acid-modified surface, potentially yielding signals below the detection threshold.(22,23) Furthermore, the specific HOMO-LUMO alignment between acetone (Figure 3f) and the amino acid frontier orbitals is critical for establishing the energy barriers and electron transfer pathways that govern the observed chirality-dependent responses.(24-26) Moreover, acetone is a polar aprotic solvent with minimal ionic conductivity, which significantly suppresses the ion transfer mechanism relative to aqueous systems, thereby supporting the electron transfer mechanism for the observed triboelectric outputs across amino acid pairs. (27,28) However, systematic exploration of solvent effects could provide a powerful means to tune sensor selectivity and sensitivity for different classes of chiral analytes, representing a promising direction for platform optimization.

Supplementary Note 3.

The high-resolution XPS analysis of the amino acid coated CuO nano-wires

The comprehensive high-resolution XPS analysis presented in Figures S4-S7 and S14-S15 demonstrates successful covalent attachment of structurally diverse chiral amino acids (L/D-arginine, L/D-cysteine, and L/D-threonine) to CuO surfaces through robust and chemically well-defined binding mechanisms. The data reveal a universal carboxylate-mediated primary binding mode, evidenced by the systematic appearance of COO^- peaks at 531-532 eV in O 1s spectra and at 288-289 eV in C 1s spectra across all amino acid modifications, coupled with the complete absence of free carboxylic acid signatures (~533 eV), confirming quantitative deprotonation and metal coordination. (29,30) This carboxylate coordination establishes the fundamental anchor point for amino acid attachment, creating thermodynamically stable metal-organic interfaces that preserve the structural integrity of both the organic modifier and the inorganic substrate. (31,32) Remarkably, all amino acids adopt zwitterionic configurations on the CuO surface, as unambiguously demonstrated by characteristic $C\text{-}NH_3^+$ peaks at 400-401 eV in the N 1s spectra, indicating either pH-dependent protonation in the local surface environment or charge compensation mechanisms following carboxylate coordination. (33,34) The side chain functionalities of each amino acid contribute distinct additional binding interactions that enhance surface attachment: arginine exhibits complex nitrogen environments reflecting its guanidinium group, (35,36) cysteine displays diagnostic S 2p signals at 162-164 eV corresponding to thiol-metal coordination that likely creates bidentate attachment modes, (37,38) and threonine shows supplementary O 1s contributions potentially arising from hydroxyl-surface hydrogen bonding interactions. (39,40) Critically, the Cu 2p spectra maintain consistent Cu $2p_3/_2$ peaks at 933-934 eV with characteristic satellite features across all modifications, confirming that the Cu^{2+} oxidation state and electronic structure of CuO remain unperturbed during ccoating, thus preserving the intrinsic properties of the metal oxide while introducing chiral functionality. (41,42) The observation of nearly identical XPS binding energies and peak profiles for L- and D-enantiomers of each amino acid indicates that the fundamental electronic binding mechanisms are stereochemically invariant, with chiral recognition properties likely emerging from the three-dimensional spatial arrangements and intermolecular interactions of surface-bound molecules rather than from detectable differences in metal-organic bond strengths. (43,44) These findings establish that amino acid coating proceeds through genuine chemical grafting rather than weak physisorption, generating robust chiral surfaces with well-defined interfacial chemistry suitable for applications demanding high stability, such as enantioselective heterogeneous catalysis, chiral separation membranes, and stereospecific molecular recognition platforms. (45,46)

Supplementary Note 4.

KPFM mapping reveals nanoscale origins of enantioselective charge transfer

Besides triboelectric output, this study focused on the use of KPFM to measure the change in surface potential following contact between chiral amino acid-coated CuO nanowires and acetone solvent. Moreover, it demonstrates an experimental technique that brings significant advances to the application of scanning-probe methods for studying enantioselective contact electrification at the molecular level. The KPFM-based approach provides two key advantages for investigating molecular recognition through contact electrification. First, multiple points on the amino acid-coated CuO nanowires can be scanned with precisely controlled surface area. Thus, it confirms the accuracy of the measurement. Second, the fast scan technique in a controlled environment allows us to scan the same nanowire region immediately before and after contact with acetone, thereby providing a point-by-point comparison of surface potential changes. By examining various surface areas before and after contact electrification, we can isolate the electronic changes arising specifically from stereospecific molecular interactions.

The KPFM results demonstrate distinct surface potential responses that correlate directly with amino acid chirality. The surface potential value of the CuO nanowire works as the baseline surface potential serving as internal controls, while the amino acid-coated areas exhibit chirality-dependent potential shifts. L-arginine-modified surfaces show surface potential changes of +25 mV following acetone contact, whereas D-arginine-coated regions display +33 mV shifts, demonstrating measurable electronic discrimination between enantiomers. Similarly, L-cysteine and D-cysteine modifications produce +14 mV and +10 mV potential changes, respectively, confirming the generality of chirality-dependent contact electrification across different amino acid structures. Moreover, the KPFM nanoscale mapping reveals that the amino acid-coated regions exhibit the mosaic surface potential distribution after the contact electrification process.

Following the contact electrification process, we identify enantioselective charge transfer patterns at the nanometer scale on amino acid-modified surfaces, where different enantiomers create distinct local electric field distributions. The observed surface potential heterogeneity (and hence the heterogeneity of transferred charge density ranging from 8.9 to 29.2 $\mu C \cdot m^{-2}$) emerges specifically from contact electrification events, rather than random surface variations or instrumental artifacts.

Supplementary Note 5.

Surface charge density calculations:

$\sigma = \varepsilon_0 \times \Delta\Phi/d$…………………………………………..(7)

$\varepsilon_0 = 8.854 \times 10^{-12}$ F·m^{-1}

d = 100×10^{-9} m = 1×10^{-7} m

For L-Arginine: $\sigma = (8.854 \times 10^{-12} \times 0.025) / (1 \times 10^{-7}) = 2.21 \times 10^{-6}$ C·m^{-2} = 22.1 μC·m^{-2}

D-Arginine: $\sigma = (8.854 \times 10^{-12} \times 0.033) / (1 \times 10^{-7}) = 2.922 \times 10^{-6}$ C·m^{-2} = 29.2 μC·m^{-2}

L-Cysteine: $\sigma = (8.854 \times 10^{-12} \times 0.014) / (1 \times 10^{-7}) = 1.240 \times 10^{-6}$ C·m^{-2} = 12.4 μC·m^{-2}

D-Cysteine: $\sigma = (8.854 \times 10^{-12} \times 0.010) / (1 \times 10^{-7}) = 8.854 \times 10^{-7}$ C·m^{-2} = 8.9 μC·m^{-2}

Supplementary Note 6.

The delocalized coordinates for the simulated orbital positions

GEOMETRY OPTIMIZATION IN DELOCALIZED COORDINATES FOR L-ARGININE

Input Coordinates (Angstroms)

ATOM		X	Y	Z
1	O	-2.419053	-2.211336	-0.310071
2	O	-3.355894	-0.859632	1.241327
3	N	-3.253662	1.358593	-0.478731
4	N	1.672444	-0.337868	0.036075
5	N	3.455148	-1.826634	0.030906
6	N	3.916679	0.423608	0.426207
7	C	-0.984079	0.432528	-0.850959
8	C	-0.381209	0.876449	0.479243
9	C	-2.501073	0.148343	-0.805421
10	C	1.140940	0.971482	0.360213
11	C	-2.789337	-0.988894	0.171904
12	C	2.934614	-0.533499	0.164141
13	H	-0.451240	-0.466959	-1.179369
14	H	-0.821750	1.201852	-1.616228
15	H	-0.798092	1.842208	0.797418
16	H	-0.622148	0.144458	1.263464
17	H	-2.797307	-0.196830	-1.806226
18	H	1.390258	1.708382	-0.429054
19	H	1.555361	1.382599	1.302574

20	H	-3.184279	1.496987	0.532914
21	H	-4.246632	1.175778	-0.631511
22	H	-2.650387	-2.839121	0.398557
23	H	2.725263	-2.440474	-0.319128
24	H	4.273528	-1.861083	-0.572164
25	H	4.651963	0.091691	1.043919
26	H	3.539944	1.307368	0.750000

Final Coordinates (Angstroms)

ATOM		X	Y	Z
1	O	-2.479832	-2.203785	-0.326942
2	O	-3.408973	-0.858923	1.236377
3	N	-3.246440	1.394800	-0.470448
4	N	1.707366	-0.372374	0.059237
5	N	3.532362	-1.810775	0.042651
6	N	3.933182	0.454148	0.421290
7	C	-1.007824	0.401060	-0.846730
8	C	-0.379002	0.837675	0.475653
9	C	-2.533097	0.160453	-0.799258
10	C	1.144943	0.931514	0.358253
11	C	-2.868021	-0.986292	0.153215
12	C	2.976198	-0.532272	0.175637
13	H	-0.497837	-0.511613	-1.179441
14	H	-0.834013	1.169109	-1.612004

15	H	-0.784988	1.809851	0.791247
16	H	-0.617954	0.112293	1.268100
17	H	-2.839668	-0.163728	-1.804502
18	H	1.388419	1.659588	-0.442084
19	H	1.553555	1.362518	1.295410
20	H	-3.179454	1.529002	0.542806
21	H	-4.244936	1.247296	-0.631887
22	H	-2.696070	-2.835006	0.385792
23	H	2.815564	-2.446702	-0.298472
24	H	4.345654	-1.824280	-0.569858
25	H	4.686975	0.146793	1.031246
26	H	3.533891	1.329650	0.744711

GEOMETRY OPTIMIZATION IN CARTESIAN COORDINATES FOR D-ARGININE

Input Coordinates (Angstroms)

ATOM		X	Y	Z
1	O	2.945302	0.454131	1.668089
2	O	4.164426	1.390979	0.016276
3	N	2.921191	0.002620	-1.947344
4	N	-1.828722	0.479416	0.465697
5	N	-3.617940	1.705627	-0.373213
6	N	-3.840349	-0.622562	-0.184621
7	C	0.961749	-0.136720	-0.469247

8	C	0.309154	-0.691645	0.796539
9	C	2.499229	-0.282283	-0.574750
10	C	-1.210402	-0.818614	0.663836
11	C	3.284295	0.623091	0.358483
12	C	-3.039144	0.497053	0.031513
13	H	0.684466	0.914237	-0.626699
14	H	0.562062	-0.679556	-1.340438
15	H	0.725909	-1.685860	1.024949
16	H	0.541036	-0.050287	1.657585
17	H	2.754062	-1.308813	-0.235323
18	H	-1.448041	-1.514352	-0.168942
19	H	-1.595793	-1.299826	1.584250
20	H	2.831760	-0.843659	-2.509949
21	H	3.905607	0.276833	-1.947464
22	H	3.532022	1.062601	2.158916
23	H	-2.980337	2.468321	-0.149812
24	H	-4.537470	1.879683	0.030624
25	H	-4.812033	-0.446594	-0.413661
26	H	-3.712039	-1.373821	0.484705

Final Coordinates (Angstroms)

ATOM		X	Y	Z
1	O	2.945302	0.454131	1.668089
2	O	4.164426	1.390979	0.016276

3	N	2.921191	0.002620	-1.947344
4	N	-1.828722	0.479416	0.465697
5	N	-3.617940	1.705627	-0.373213
6	N	-3.840349	-0.622562	-0.184621
7	C	0.961749	-0.136720	-0.469247
8	C	0.309154	-0.691645	0.796539
9	C	2.499229	-0.282283	-0.574750
10	C	-1.210402	-0.818614	0.663836
11	C	3.284295	0.623091	0.358483
12	C	-3.039144	0.497053	0.031513
13	H	0.684466	0.914237	-0.626699
14	H	0.562062	-0.679556	-1.340438
15	H	0.725909	-1.685860	1.024949
16	H	0.541036	-0.050287	1.657585
17	H	2.754062	-1.308813	-0.235323
18	H	-1.448041	-1.514352	-0.168942
19	H	-1.595793	-1.299826	1.584250
20	H	2.831760	-0.843659	-2.509949
21	H	3.905607	0.276833	-1.947464
22	H	3.532022	1.062601	2.158916
23	H	-2.980337	2.468321	-0.149812
24	H	-4.537470	1.879683	0.030624
25	H	-4.812033	-0.446594	-0.413661
26	H	-3.712039	-1.373821	0.484705

GEOMETRY OPTIMIZATION IN DELOCALIZED COORDINATES FOR L-CYSTEINE

Input Coordinates (Angstroms)

ATOM		X	Y	Z
1	S	2.388945	1.179699	-0.169257
2	O	-2.055987	0.794890	0.705346
3	O	-0.854149	1.468517	-1.093395
4	N	-0.623107	-1.615114	0.222540
5	C	0.030116	-0.299955	0.366929
6	C	1.313386	-0.279111	-0.464484
7	C	-0.964381	0.754028	-0.116538
8	H	0.287512	-0.029846	1.411788
9	H	1.934255	-1.140658	-0.177442
10	H	1.085761	-0.372886	-1.533094
11	H	-1.481735	-1.639415	0.776630
12	H	-0.006846	-2.336699	0.602279
13	H	1.605753	2.055046	-0.848861
14	H	-2.659524	1.461505	0.317559

Final Coordinates (Angstroms)

ATOM		X	Y	Z
1	S	2.032177	1.254006	-0.366676
2	O	-1.520602	0.315157	0.028232
3	O	-0.041997	1.199445	-0.484638

4	N	-0.960476	-1.474156	0.625792
5	C	-0.038209	-0.329225	0.750568
6	C	0.858257	-0.153873	-0.475902
7	C	-0.562669	1.098621	0.608811
8	H	0.911756	0.240900	0.809803
9	H	1.501385	-1.041027	-0.572932
10	H	0.256415	-0.076282	-1.389277
11	H	-1.352029	-1.706377	1.541001
12	H	-0.434731	-2.293287	0.313870
13	H	1.096517	2.215832	-0.568931
14	H	-1.745794	0.750264	-0.819723

GEOMETRY OPTIMIZATION IN DELOCALIZED COORDINATES FOR D-CYSTEINE

Input Coordinates (Angstroms)

ATOM		X	Y	Z
1	S	1.975052	-1.511632	-0.070098
2	O	-1.633189	-1.443365	0.784478
3	O	-1.461051	-0.780793	-1.378240
4	N	-0.734134	1.687240	0.028458
5	C	-0.140025	0.373449	0.298878
6	C	1.235440	0.150961	-0.358453
7	C	-1.134164	-0.669574	-0.210675
8	H	-0.049488	0.259869	1.389037

9	H	1.132387	0.220213	-1.451291
10	H	1.936553	0.931152	-0.030578
11	H	-0.865631	1.803480	-0.979668
12	H	-0.108522	2.429032	0.347292
13	H	2.099760	-1.381107	1.276654
14	H	-2.252989	-2.068926	0.354205

Final Coordinates (Angstroms)

	ATOM	X	Y	Z
1	S	1.973613	-1.512593	-0.070887
2	O	-1.628254	-1.447197	0.784887
3	O	-1.464781	-0.776788	-1.376207
4	N	-0.733212	1.688385	0.027570
5	C	-0.140249	0.375296	0.299683
6	C	1.235279	0.151084	-0.357272
7	C	-1.133762	-0.668276	-0.209519
8	H	-0.049969	0.262258	1.389884
9	H	1.132966	0.221568	-1.449942
10	H	1.937255	0.929855	-0.028345
11	H	-0.868900	1.801876	-0.980118
12	H	-0.105972	2.430494	0.341878
13	H	2.093529	-1.383897	1.276132
14	H	-2.247545	-2.072067	0.352255

GEOMETRY OPTIMIZATION IN DELOCALIZED COORDINATES FOR L-THHREONINE

Input Coordinates (Angstroms)

ATOM		X	Y	Z
1	O	-1.585200	-1.303243	0.611251
2	O	1.147211	-1.866827	0.149701
3	O	2.383405	-0.023931	-0.520202
4	N	0.612751	1.748405	0.821269
5	C	-1.095177	0.018932	0.202317
6	C	0.325145	0.323493	0.819385
7	C	-1.152083	0.214089	-1.317071
8	C	1.410199	-0.479131	0.098288
9	H	0.267687	-0.040151	1.858561
10	H	-1.768614	0.728427	0.698435
11	H	-0.868560	1.243424	-1.590509
12	H	-2.178143	0.033138	-1.667506
13	H	-0.482597	-0.485517	-1.846309
14	H	1.067927	2.115395	-0.015080
15	H	0.980790	2.137472	1.687316
16	H	-0.912769	-1.992359	0.358547
17	H	1.848029	-2.371616	-0.348392

Final Coordinates (Angstroms)

	ATOM	X	Y	Z
1	O	-1.578320	-1.308069	0.605548
2	O	1.149223	-1.868468	0.160192
3	O	2.386675	-0.028832	-0.516437
4	N	0.614533	1.748698	0.816354
5	C	-1.093799	0.017582	0.201594
6	C	0.326367	0.324019	0.818641
7	C	-1.157803	0.218579	-1.316814
8	C	1.412632	-0.481547	0.102746
9	H	0.267877	-0.036676	1.858813
10	H	-1.768075	0.723066	0.702243
11	H	-0.877109	1.249440	-1.587666
12	H	-2.185139	0.037620	-1.663414
13	H	-0.490283	-0.478538	-1.851603
14	H	1.074681	2.112223	-0.018761
15	H	0.976804	2.141433	1.683117
16	H	-0.907581	-1.994606	0.341609
17	H	1.849316	-2.375924	-0.336163

GEOMETRY OPTIMIZATION IN DELOCALIZED COORDINATES FOR D-THREONINE

Input Coordinates (Angstroms)

ATOM		X	Y	Z
1	O	0.499952	0.533522	1.547533
2	O	-2.155149	1.300142	-0.547656
3	O	-2.717508	-0.604042	0.649696
4	N	-0.133671	-1.764569	0.097256
5	C	0.586454	0.592633	0.066702
6	C	-0.466413	-0.444222	-0.401314
7	C	1.994717	0.272499	-0.432818
8	C	-1.881706	0.030733	-0.009335
9	H	-0.437709	-0.448199	-1.505498
10	H	0.261535	1.590566	-0.274397
11	H	2.725019	0.992817	-0.025788
12	H	2.040275	0.343411	-1.532209
13	H	2.274420	-0.748050	-0.136215
14	H	-0.040881	-1.796171	1.115310
15	H	-0.690452	-2.531888	-0.279514
16	H	1.211918	1.087929	1.953358
17	H	-3.070800	1.592889	-0.285111

Final Coordinates (Angstroms)

ATOM		X	Y	Z
1	O	0.499124	0.529816	1.546264
2	O	-2.151130	1.304480	-0.540338

3	O	-2.720783	-0.608416	0.638459
4	N	-0.132172	-1.765168	0.098987
5	C	0.585879	0.591630	0.065588
6	C	-0.466026	-0.445234	-0.402610
7	C	1.994765	0.272846	-0.433899
8	C	-1.881240	0.030358	-0.011799
9	H	-0.436699	-0.451993	-1.506945
10	H	0.260695	1.590046	-0.273630
11	H	2.724371	0.993789	-0.026925
12	H	2.040286	0.343923	-1.533201
13	H	2.276100	-0.747403	-0.137964
14	H	-0.037776	-1.791906	1.117222
15	H	-0.694030	-2.531534	-0.272482
16	H	1.206540	1.089329	1.952945
17	H	-3.067904	1.595436	-0.279671

No.	Chiral molecule detection technique	Real-time monitoring	Response time	Requirement of trained personnel / complicated sample preparation	Label-free detection	Ref.
**1**	**Charge transfer via contact electrification**	**Yes**	**500 (ms)**	**No**	**Yes**	**This work**
**2**	Fluorescence analysis	Yes	< 1 (min)	Yes	No	(47)
**3**	Microbubble-induced accumulation-assisted plasmonic chiral sensing	No	1 (min)	Yes	Yes	(48)
**4**	SERS-based enantioselective detection	No	100 (s)	Yes	No	(49)
**5**	Enantioselective interaction between chiral Molecules and chiral high-Miller index facets	No	2 (min)	Yes	No	(50)
**6**	pH-triggered colorimetric strategy for enantioselective discrimination	No	5 (min)	Yes	No	(51)
**7**	Imprinted polymer-based SERS	No	10 (min)	No	No	(52)
**8**	Enantioselective detection	No	15 (min)	Yes	No	(53)
**9**	Enzyme-free SERS	No	30 (min)	Yes	Yes	(54)
**10**	Fluorescence analysis	No	30 (min)	Yes	No	(55)

Table S1: Comparative analysis of chiral molecule detection techniques.

No	Amino Acid	Degradation Percentage (%)
**1.**	L-Arginine	45.0%
**2.**	D-Arginine	51.7%
**3.**	L-Cysteine	20.0%
**4.**	D-Cysteine	18.3%
**5.**	L-Threonine	36.7%
**6.**	D-Threonine	38.3%

Table S2: Methyl orange degradation percentages.

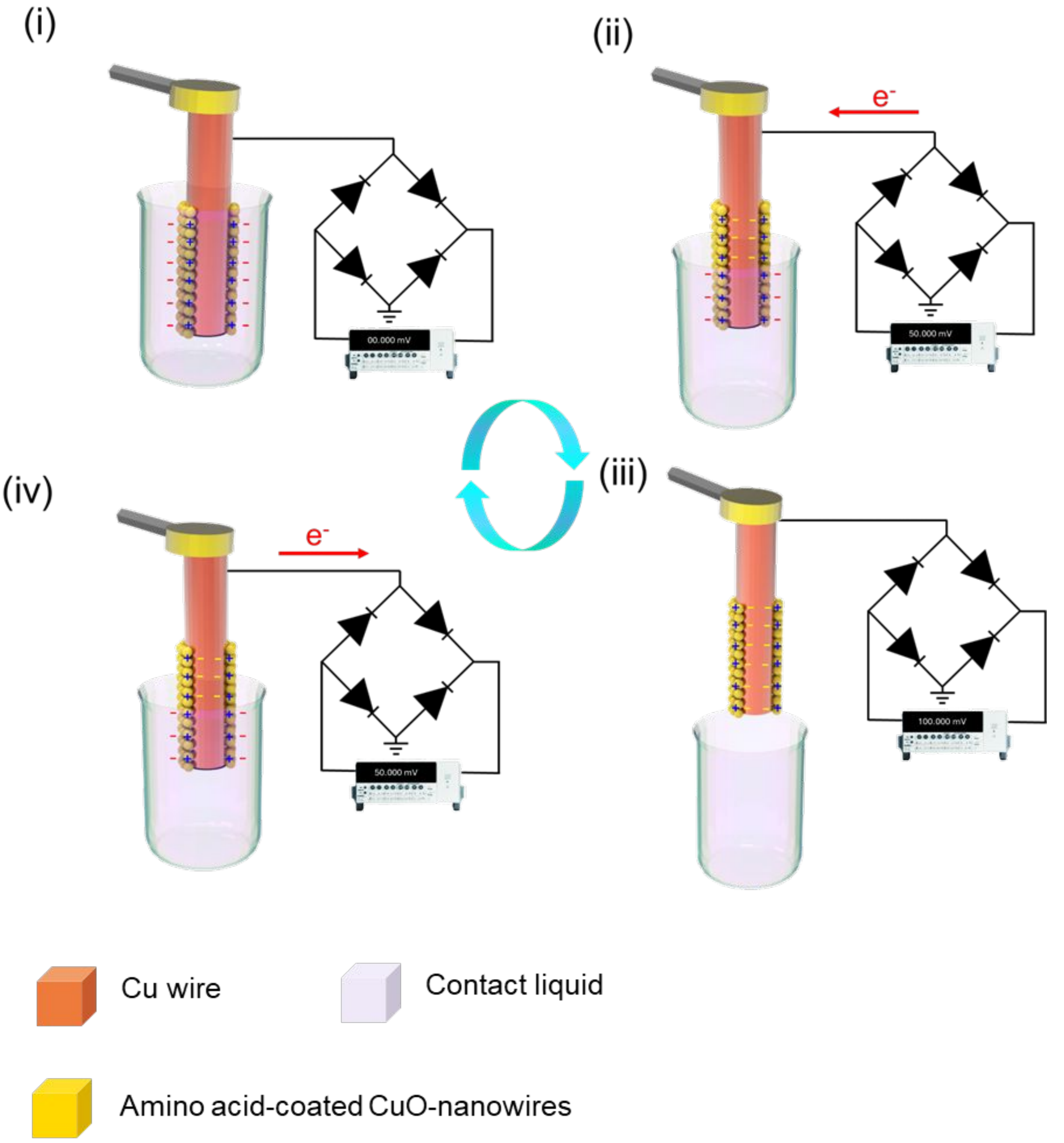

Figure S1. Working mechanism illustration of the CuO-nanowire-based solid-liquid TENG. (i) When the solid friction layer is immersed in the contact liquid, contact electrification causes the solid surface to acquire positive charges, while the liquid surface acquires negative charges. (ii)–(iii) As the separation process proceeds, the generated potential induces electron flow from the ground to the Cu wire until equilibrium is achieved. The induced negative charges on the Cu electrode are shown in blue. (iv) When the solid friction layer begins to immerse in the liquid again, the reduction in potential difference drives electron flow back from the Cu wire to the ground until the potential difference returns to zero, restoring the original state (i).

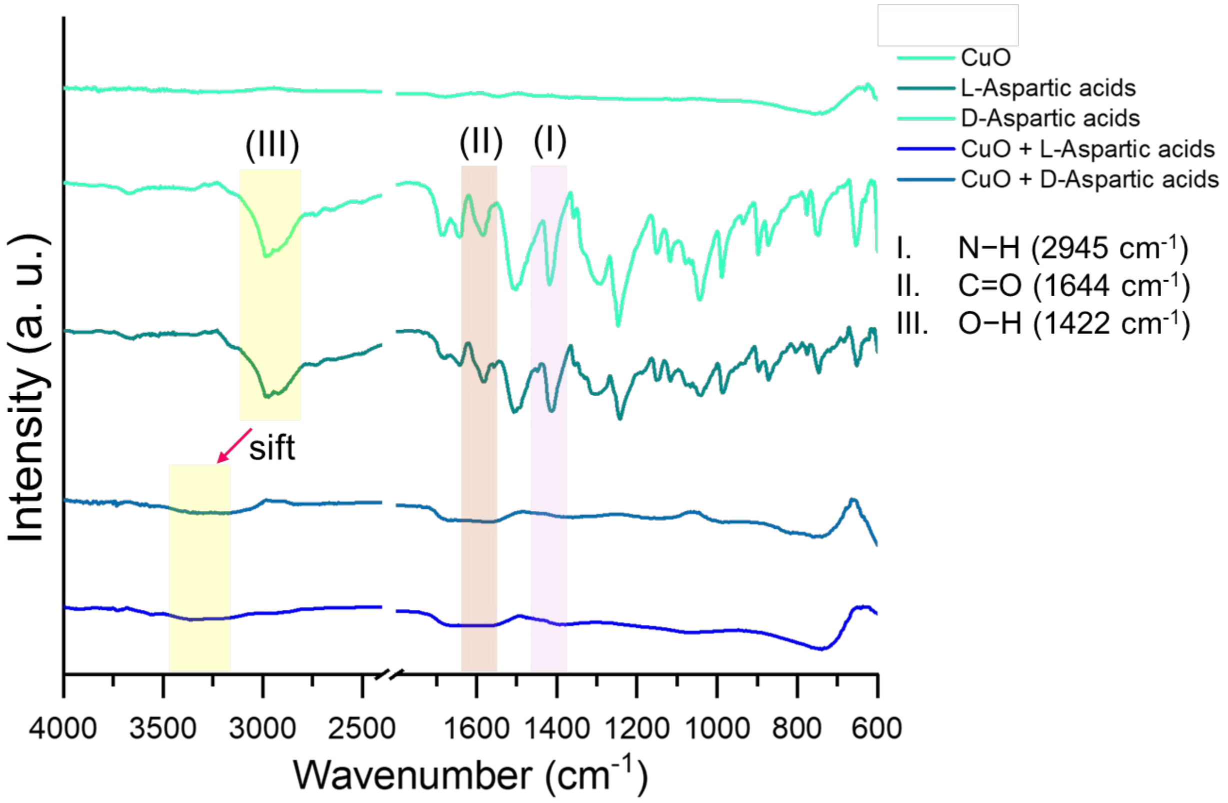

Figure S2. FTIR spectra analysis of CuO before and after chiral amino acid coating. The spectra show pure CuO, L-aspartic acid, D-aspartic acid, CuO + L-aspartic acid composite, and CuO + D-aspartic acid composite. Three key spectral regions are highlighted: (I) N-H stretching vibrations at 2945 cm^{-1}, (II) C=O stretching vibrations at 1644 cm^{-1}, and (III) O-H bending vibrations at 1422 cm^{-1}. The presence of these characteristic peaks in the composite materials confirms successful coating of CuO with chiral amino acids. A notable shift in the O-H bending region is observed upon attachment of the amino acid, indicating a chemical interaction between the CuO surface and the chiral molecules.

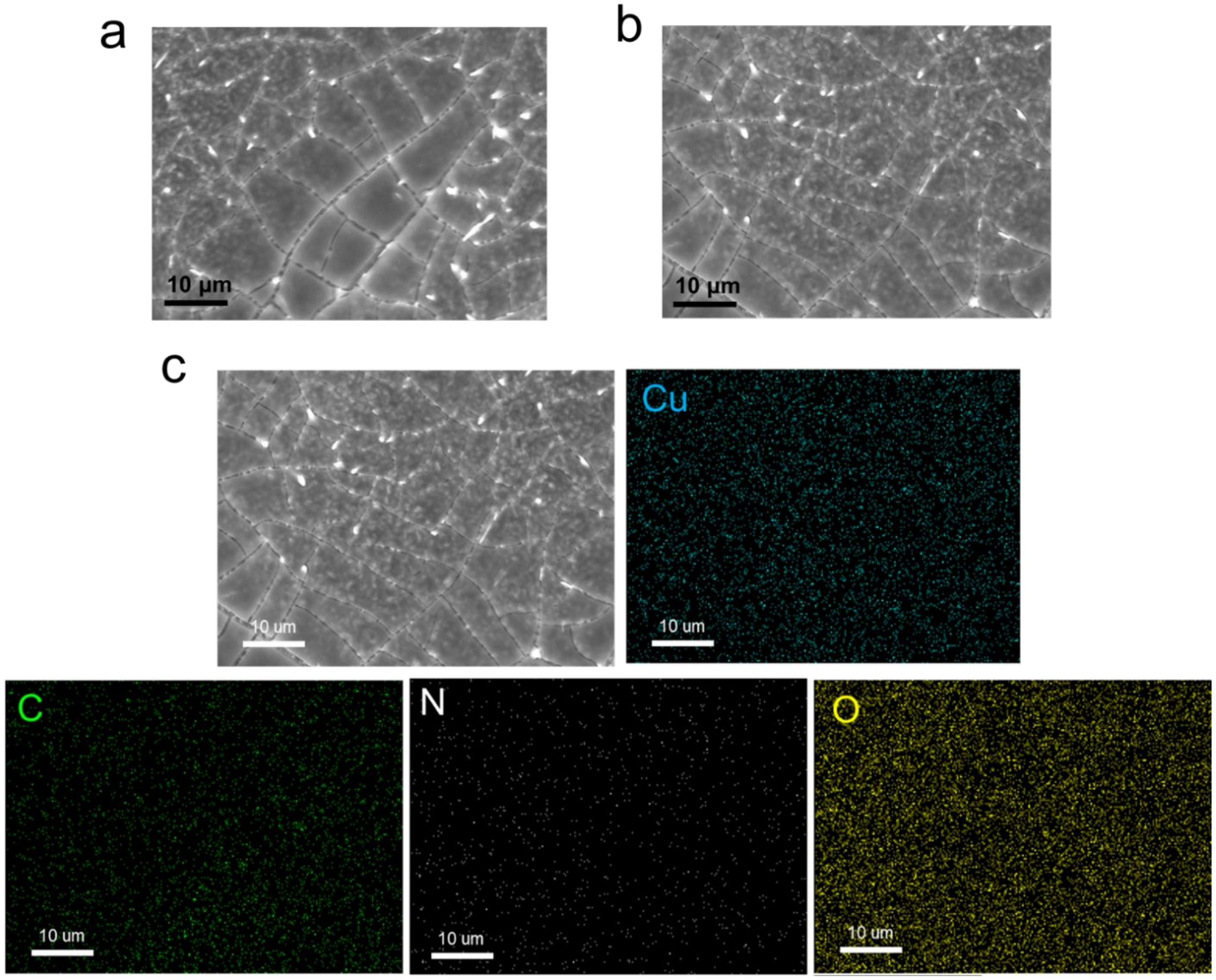

Figure S3. SEM and EDS characterization of the CuO nanowires modified with chiral amino acids. SEM images showing the morphology of (a) CuO + L-aspartic acid and (b) CuO + D-aspartic acid composites, revealing that the nanowires are covered by the amino acids uniformly. (c) Representative SEM image and corresponding EDS elemental mapping showing the uniform distribution of Cu (cyan), C (green), N (white), and O (yellow) elements across the modified CuO nanowires. The presence of carbon and nitrogen signals confirms successful attachment of aspartic acid molecules to the CuO surface, while the homogeneous distribution indicates uniform coating of the chiral amino acids on the nanowire structure. Scale bars: 10 µm.

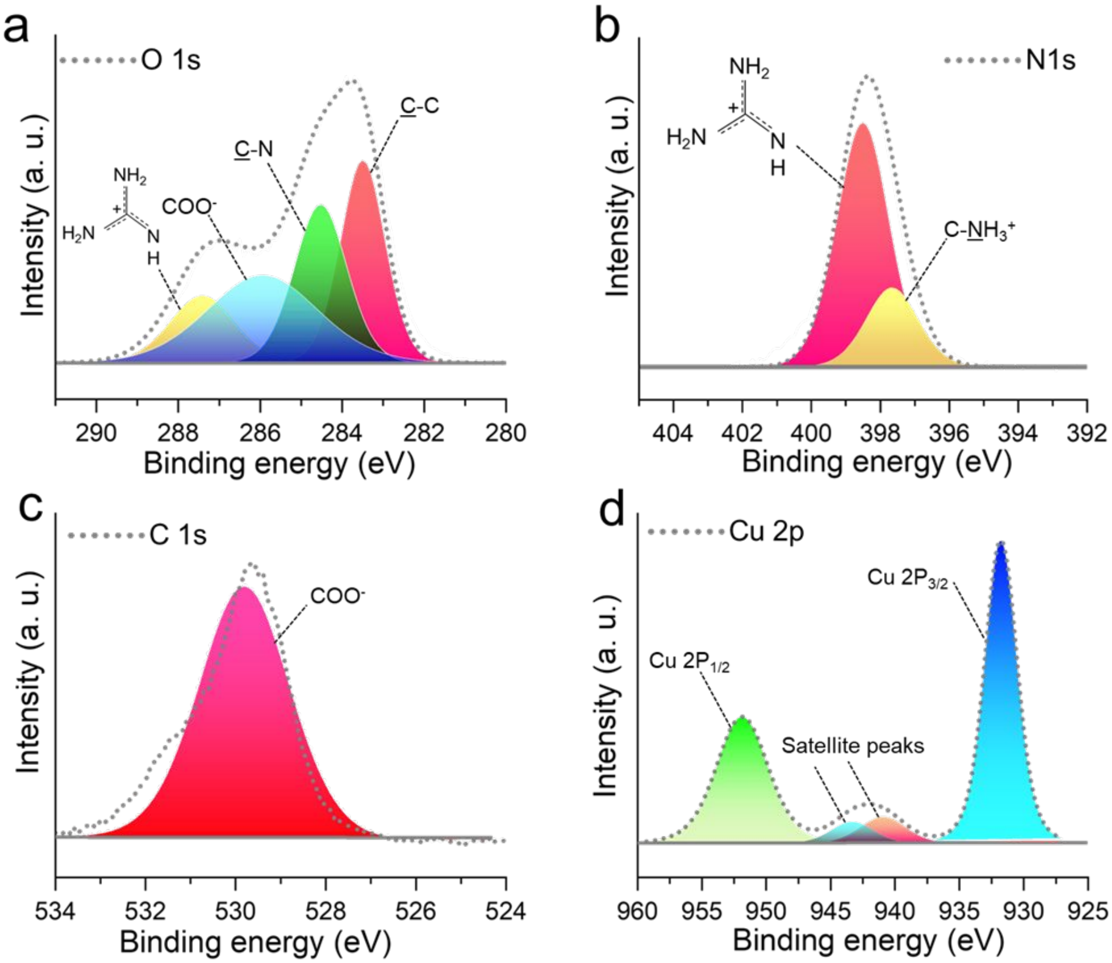

Figure S4. High-resolution XPS analysis of CuO modified with L-arginine. High-resolution XPS spectra showing the chemical composition and bonding states after chiral amino acid modification: (a) O 1s spectrum with deconvoluted peaks corresponding to COO^- groups, C-N bonds, and C-C bonds from the attached L-arginine molecules; (b) N 1s spectrum displaying peaks attributed to NH_2 groups and protonated C-NH_3^+ species, confirming the presence of amino acid functionalities; (c) C 1s spectrum showing the characteristic COO^- peak from the carboxylate groups of arginine; and (d) Cu 2p spectrum exhibiting Cu $2p_3/_2$ and Cu $2p_1/_2$ peaks with their satellite features, indicating the Cu^{2+} oxidation state in CuO is maintained after amino acid attachment. The XPS results provide direct evidence for successful covalent bonding between L-arginine and the CuO surface.

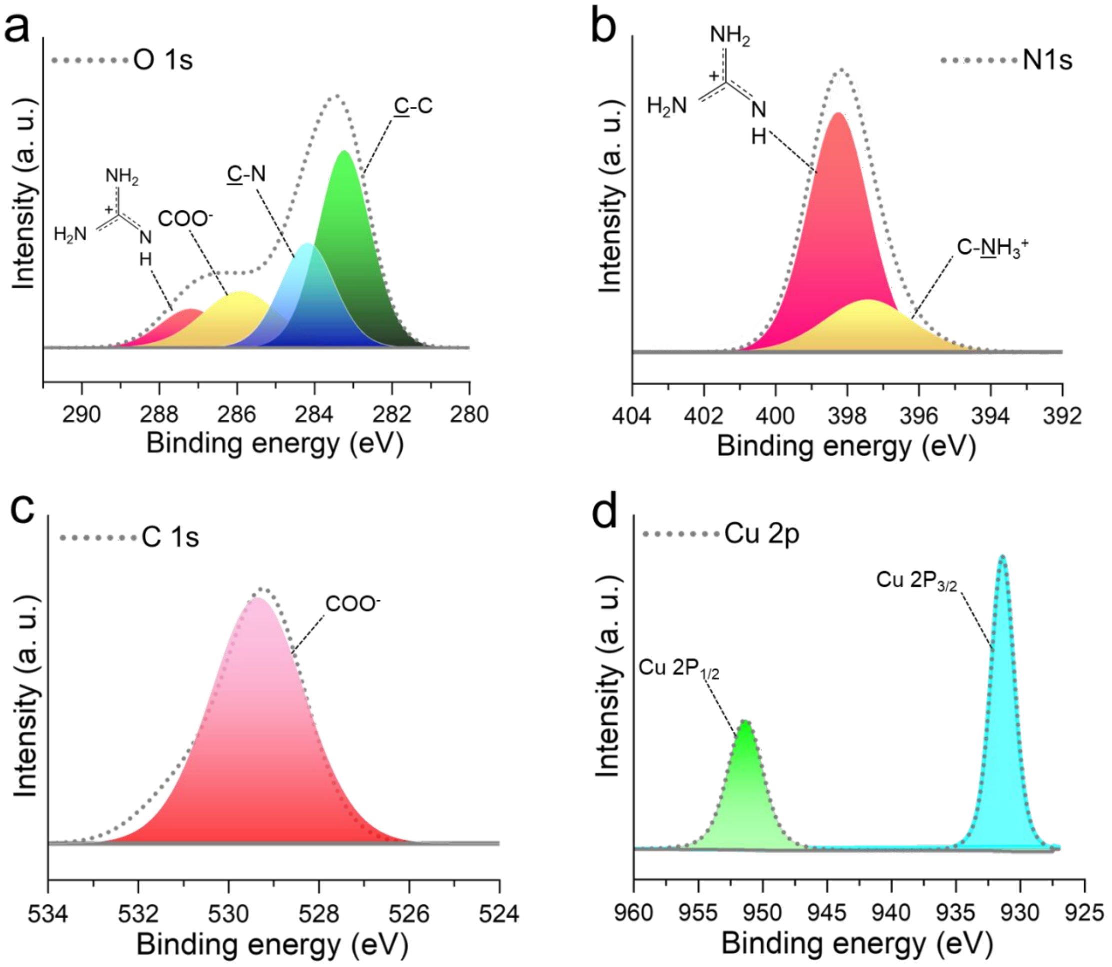

Figure S5. High-resolution XPS analysis of CuO modified with D-arginine. High-resolution XPS spectra showing the chemical composition and bonding states after chiral amino acid modification: (a) O 1s spectrum with deconvoluted peaks corresponding to COO^- groups, C-N bonds, and C-C bonds from the attached D-arginine molecules; (b) N 1s spectrum displaying peaks attributed to NH_2 groups and protonated $C\text{-}NH_3^+$ species, confirming the presence of amino acid functionalities; (c) C 1s spectrum showing the characteristic COO^- peak from the carboxylate groups of arginine; and (d) Cu 2p spectrum exhibiting Cu $2p_3/_2$ and Cu $2p_1/_2$ peaks, indicating the Cu^{2+} oxidation state in CuO is maintained after amino acid attachment. The XPS results provide direct evidence for successful covalent bonding between D-arginine and the CuO surface, with similar binding characteristics to the L-enantiomer but maintaining the distinct chiral configuration.

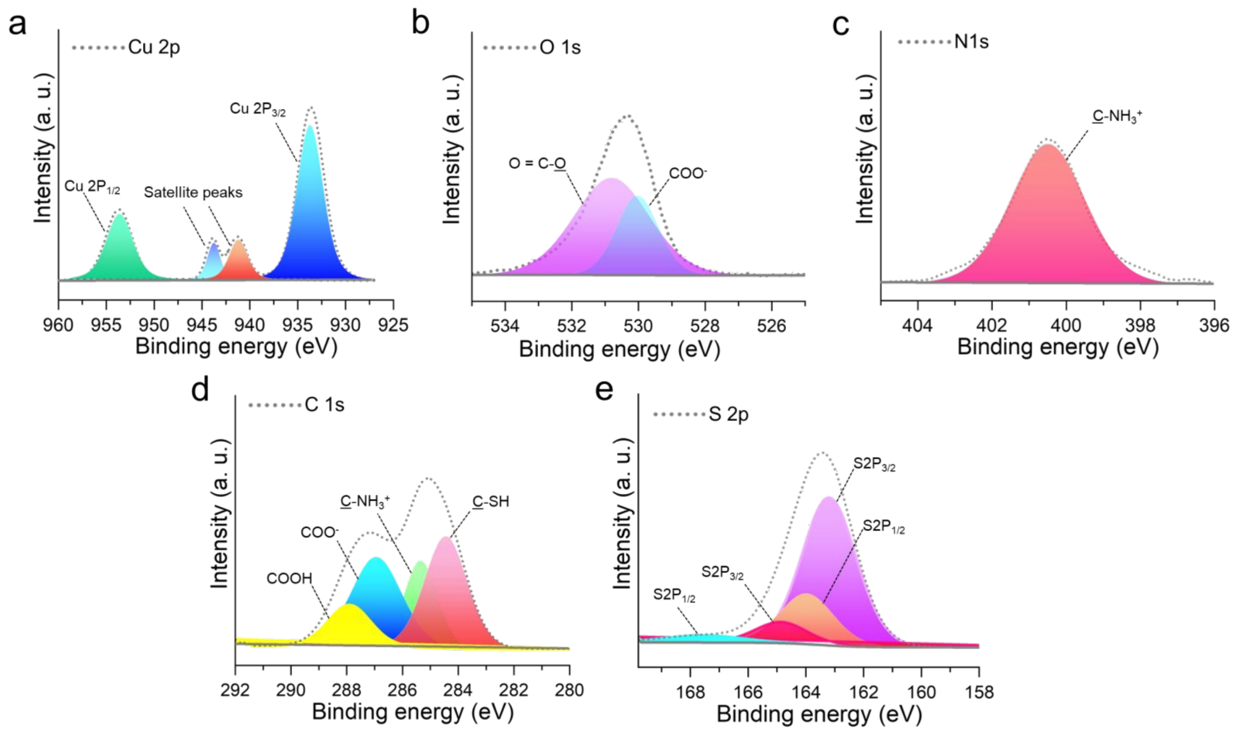

Figure S6. High-resolution XPS analysis of CuO modified with L-cysteine. High-resolution XPS spectra showing the chemical composition and bonding states after chiral amino acid modification: (a) Cu 2p spectrum exhibiting Cu $2p_3/_2$ and Cu $2p_1/_2$ peaks with satellite features, confirming the Cu^{2+} oxidation state is preserved; (b) O 1s spectrum with deconvoluted peaks corresponding to O=C-O and COO^- groups from the attached L-cysteine molecules; (c) N 1s spectrum displaying the characteristic C-NH_3^+ peak, indicating protonated amino groups; (d) C 1s spectrum showing multiple carbon environments including COOH, COO^-, C-NH_3^+, and C-SH bonds from the cysteine structure; and (e) S 2p spectrum revealing $S2P_3/_2$ and $S2P_1/_2$ doublets, confirming the presence of thiol (-SH) groups from cysteine.

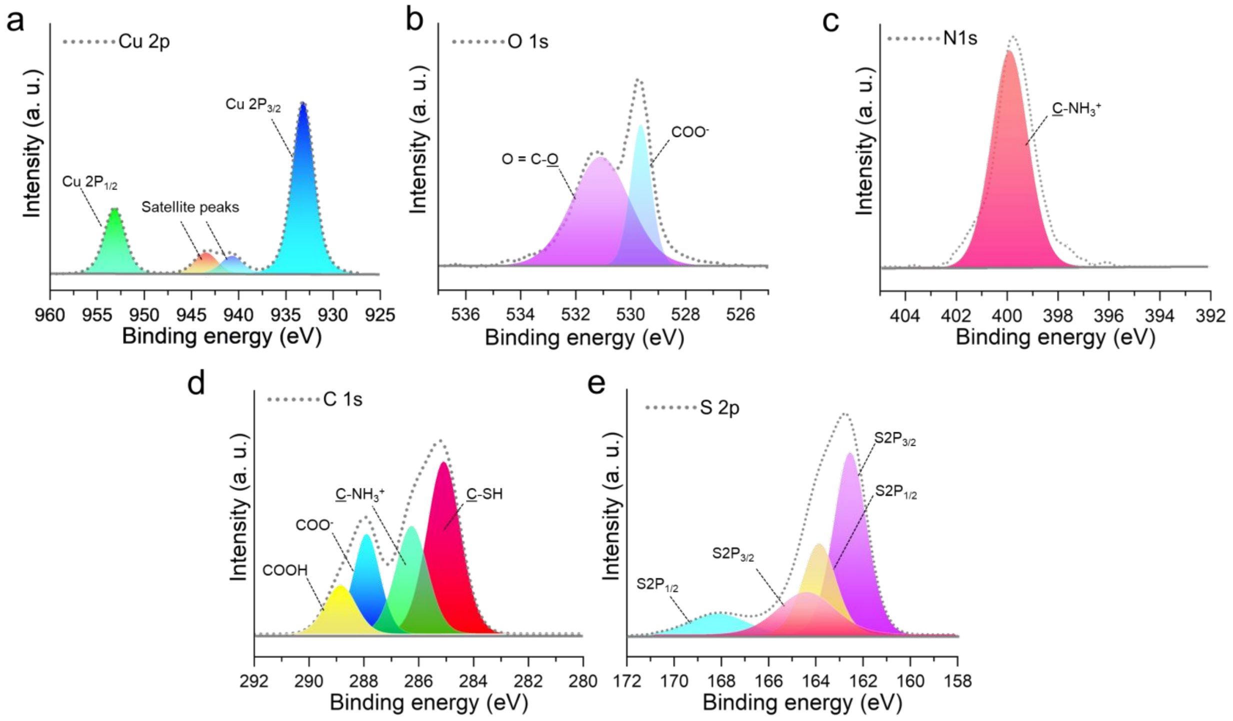

Figure S7. High-resolution XPS analysis of CuO modified with D-cysteine. High-resolution XPS spectra showing the chemical composition and bonding states after chiral amino acid modification: (a) Cu 2p spectrum exhibiting Cu $2p_3/_2$ and Cu $2p_1/_2$ peaks with satellite features, confirming the Cu^{2+} oxidation state is maintained; (b) O 1s spectrum with deconvoluted peaks corresponding to O=C-O and COO^- groups from the attached D-cysteine molecules; (c) N 1s spectrum displaying the characteristic C-NH_3^+ peak, indicating protonated amino groups; (d) C 1s spectrum showing multiple carbon environments including COOH, COO^-, C-NH_3^+, and C-SH bonds from the cysteine structure; and (e) S 2p spectrum revealing $S2P_3/_2$ and $S2P_1/_2$ doublets, confirming the presence of thiol (-SH) groups from cysteine. The XPS results provide direct evidence for successful attachment of D-cysteine to the CuO surface, with similar binding characteristics to the L-enantiomer while maintaining the distinct chiral configuration through the thiol-metal interaction.

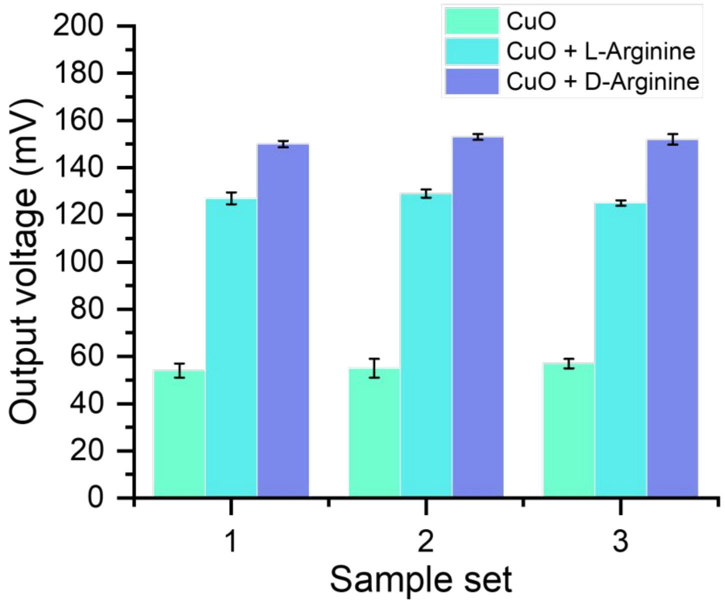

Figure S8. Statistical comparison of output voltages across three sample sets demonstrating consistent performance differences for L/D- arginine detection.

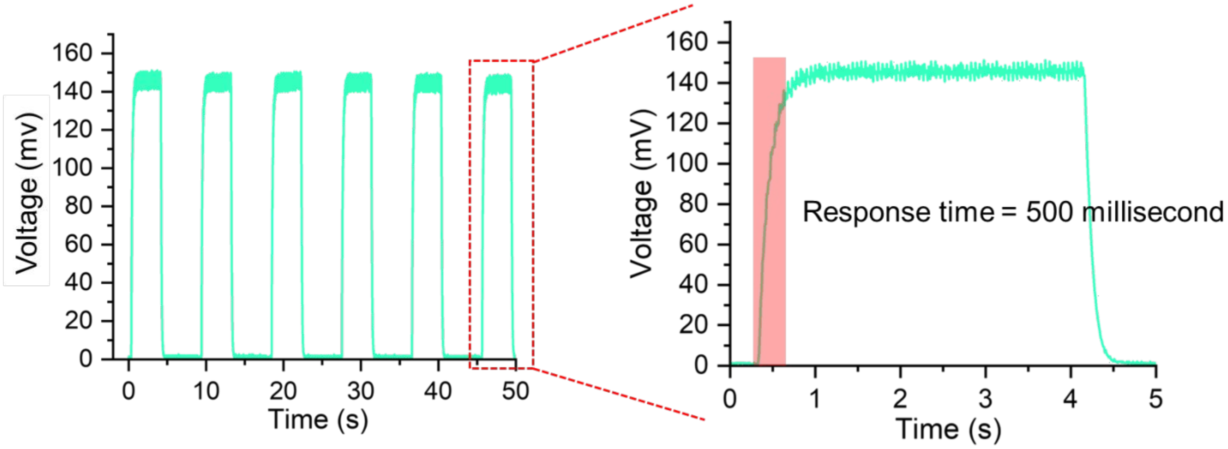

Figure S9. Electrical response characteristics of the chiral amino acid-modified CuO TENG. The response time measurement reveals a fast response time of 500 milliseconds (highlighted in pink) for the voltage to reach its maximum value upon contact, followed by a stable output plateau and rapid decay upon separation. The excellent stability and fast response demonstrate the robust performance of the chiral-modified TENG for practical applications.

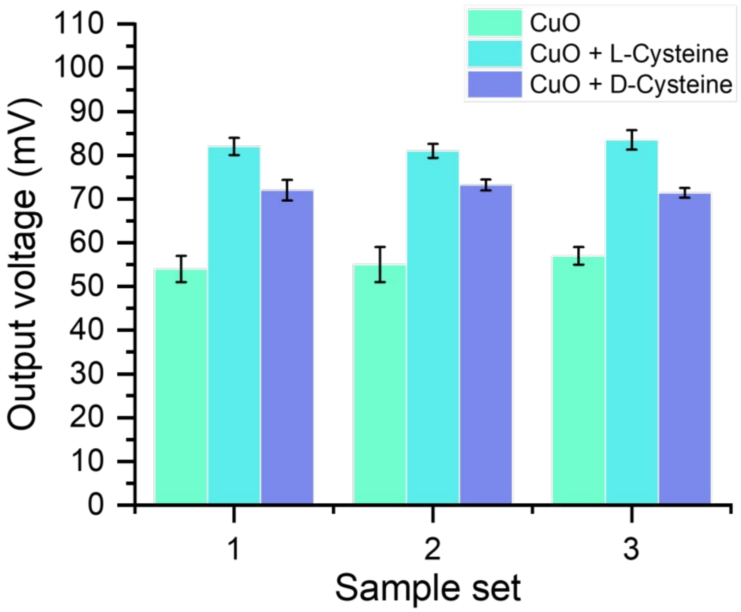

Figure S10. Statistical comparison of output voltages across three sample sets demonstrating consistent performance differences for L/D- cysteine detection.

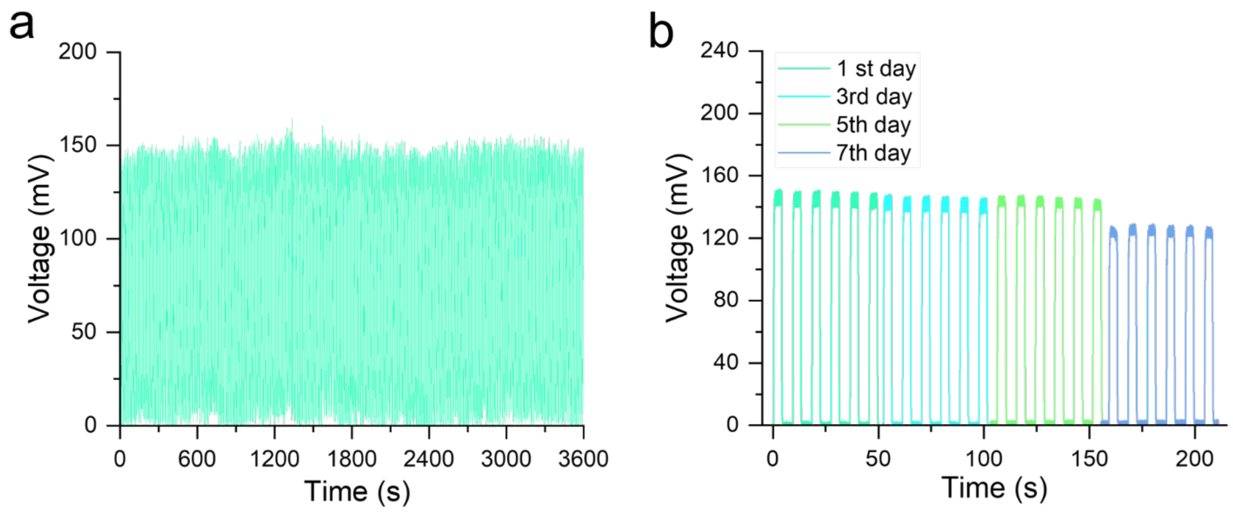

Figure S11: Long-term stability and reusability of D-Arginine modified sensor (a) Long-term stability test over 3600 seconds (1 hour), demonstrating stable output, and (b) reusability test of the voltage output displays the reusability of the sensor across multiple operational cycles until 5th day.

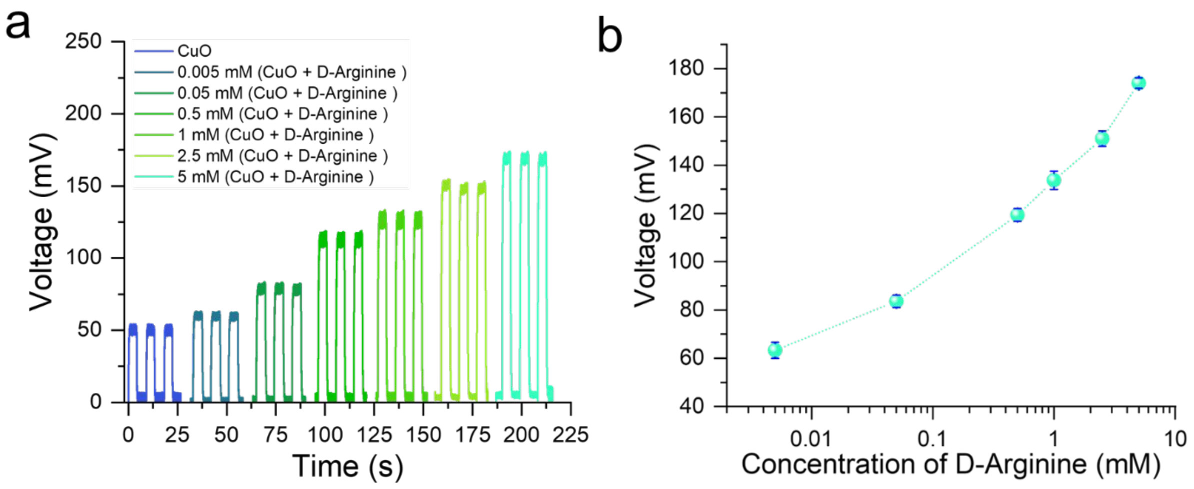

Figure. S12. Quantitative concentration-dependent sensor performance and analytical metrics. (a) Real-time voltage output profiles of CuO nanowires coated with D-arginine at concentrations ranging from 0.005 mM to 5 mM, demonstrating consistent signal generation across three orders of magnitude with baseline CuO control. (b) Calibration curve showing a linear relationship between D-arginine concentration and triboelectric voltage output.

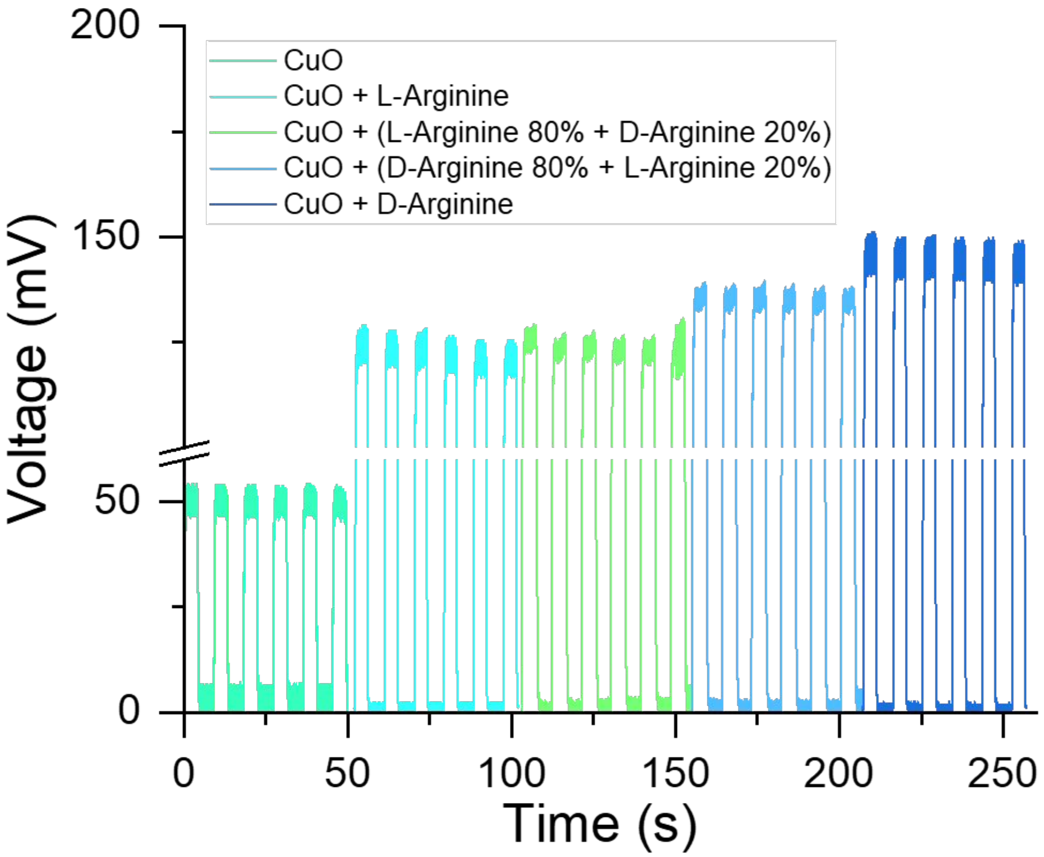

Figure S13. Selectivity test of the sensor using racemic mixtures of L-/D-arginine enantiomers.

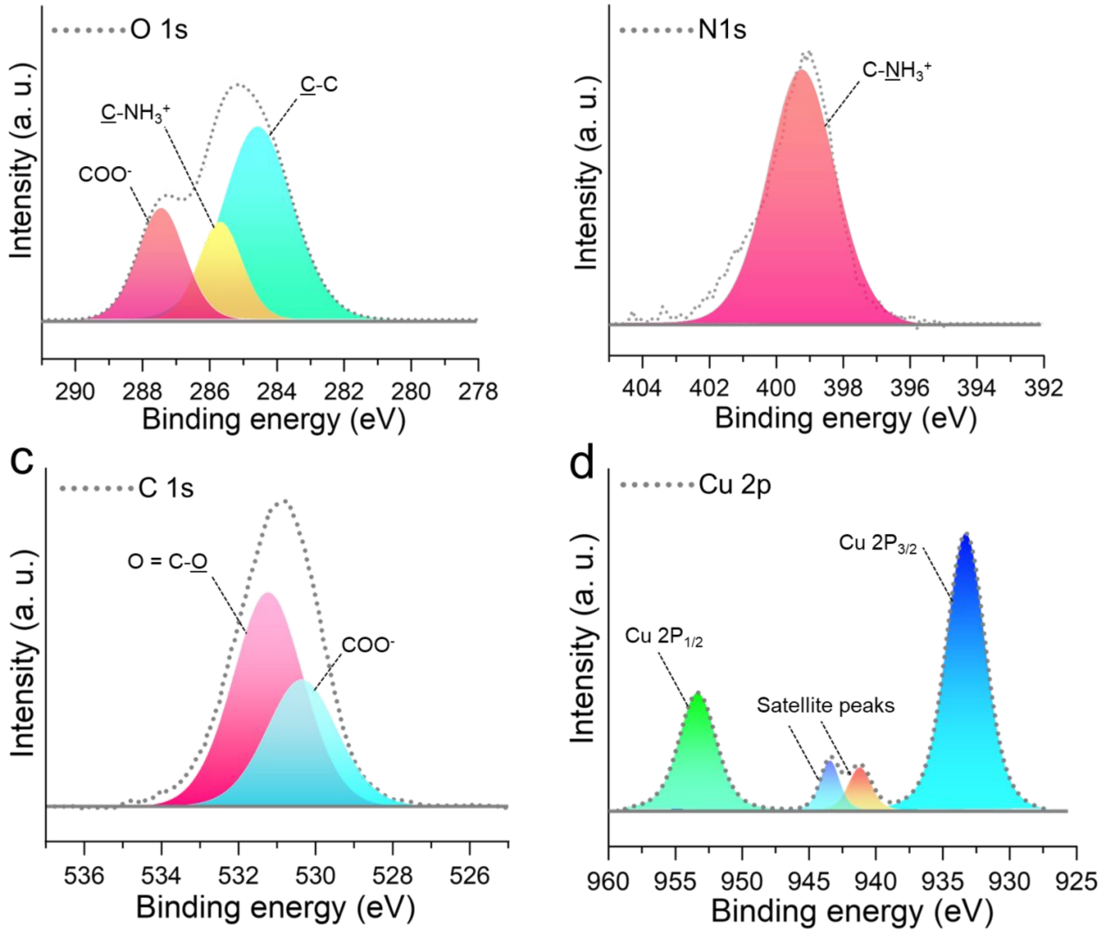

Figure S14: High-resolution XPS analysis of CuO modified with L-threonine. High-resolution XPS spectra showing the chemical composition and bonding states after chiral amino acid modification: (a) O 1s spectrum with deconvoluted peaks corresponding to COO^- groups, C-NH_3^+ bonds, and C-C bonds from the attached L-threonine molecules; (b) N 1s spectrum displaying the characteristic C-NH_3^+ peak, confirming the presence of protonated amino groups from threonine; (c) C 1s spectrum showing peaks attributed to O=C-O and COO^- groups from the carboxylate functionalities of threonine; and (d) Cu 2p spectrum exhibiting Cu $2p_3/_2$ and Cu $2p_1/_2$ peaks with satellite features, indicating the Cu^{2+} oxidation state in CuO is preserved after amino acid attachment. The XPS results provide direct evidence for successful covalent bonding between L-threonine and the CuO surface, demonstrating the versatility of the chiral coating approach with hydroxyl-containing amino acids.

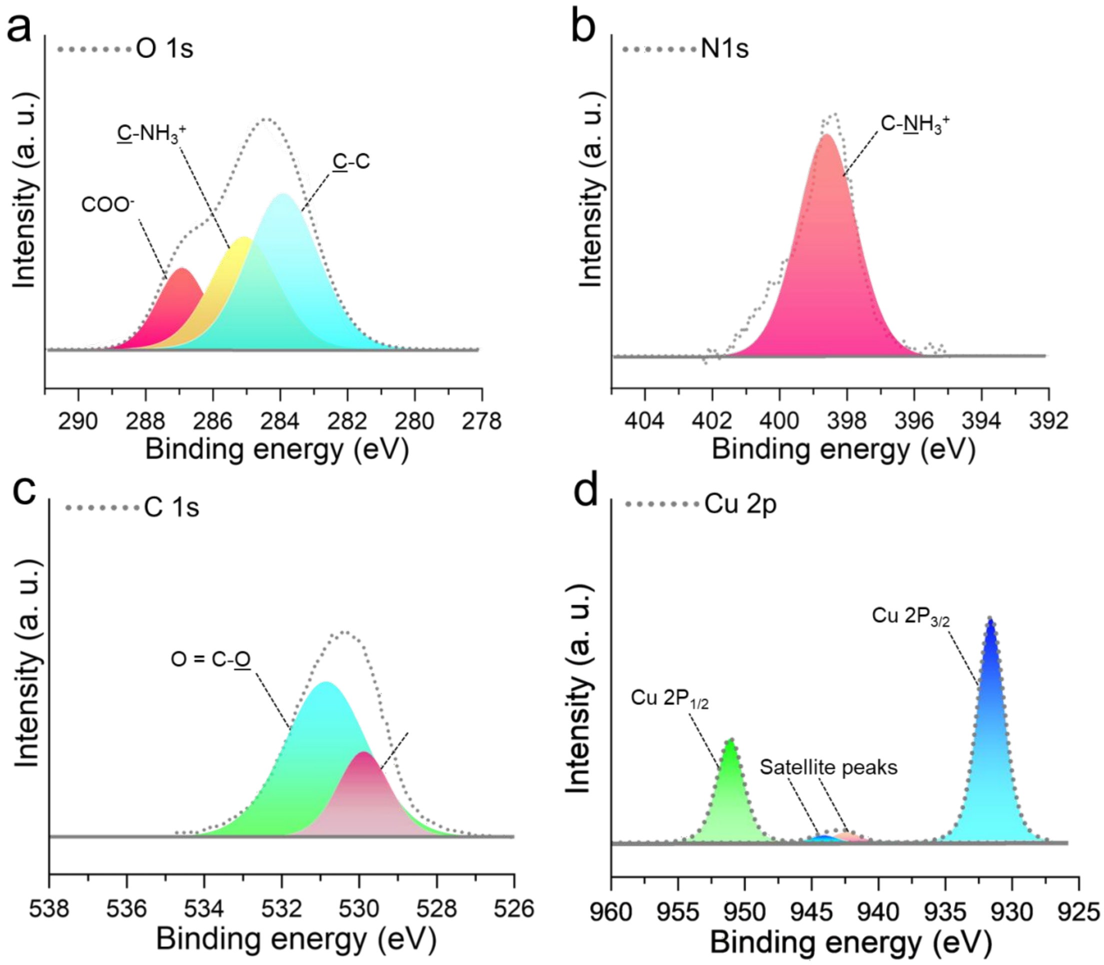

Figure S15. High-resolution XPS analysis of CuO modified with D-threonine. High-resolution XPS spectra showing the chemical composition and bonding states after chiral amino acid modification: (a) O 1s spectrum with deconvoluted peaks corresponding to COO^- groups, $C\text{-}NH_3^+$ bonds, and C-C bonds from the attached D-threonine molecules; (b) N 1s spectrum displaying the characteristic $C\text{-}NH_3^+$ peak, confirming the presence of protonated amino groups from threonine; (c) C 1s spectrum showing the peak attributed to O=C-O groups from the carboxylate functionalities of threonine; and (d) Cu 2p spectrum exhibiting Cu $2p_3/_2$ and Cu $2p_1/_2$ peaks with satellite features, indicating the Cu^{2+} oxidation state in CuO is preserved after amino acid attachment. The XPS results provide direct evidence for successful covalent bonding between D-threonine and the CuO surface, with similar binding characteristics to the L-enantiomer while maintaining the distinct chiral configuration of the D-form amino acid.

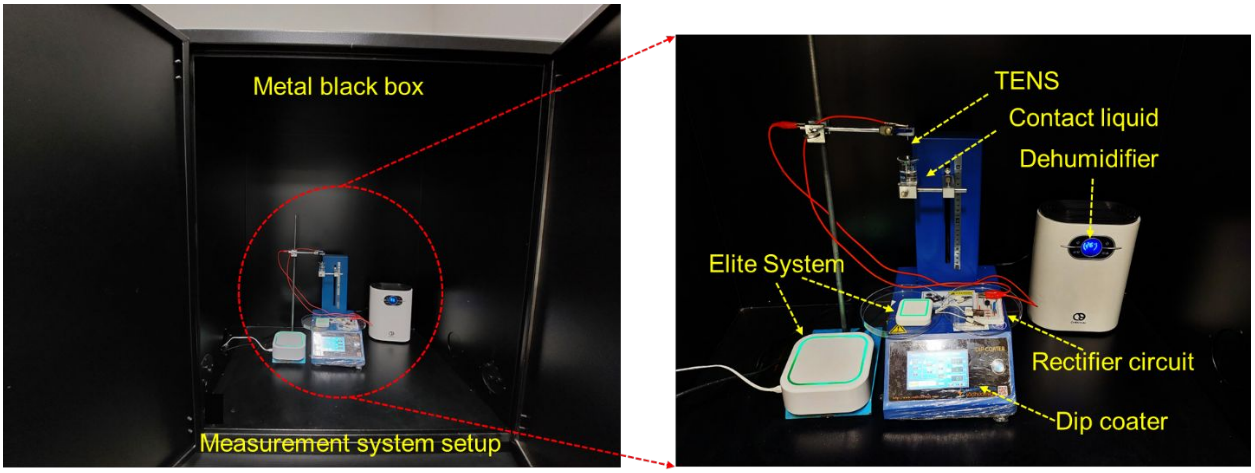

Figure S16. The digital image of the solid-liquid contact electrification-based homochiral amino acid discrimination and detection system.

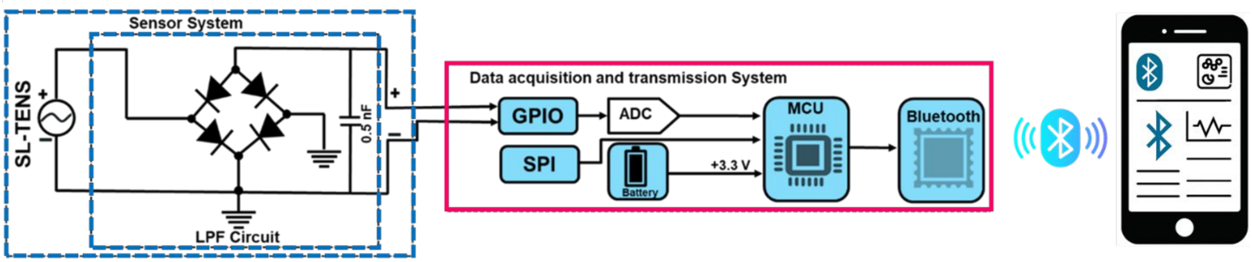

Figure S17. Circuit diagram of the wireless data transmission and acquisition process.

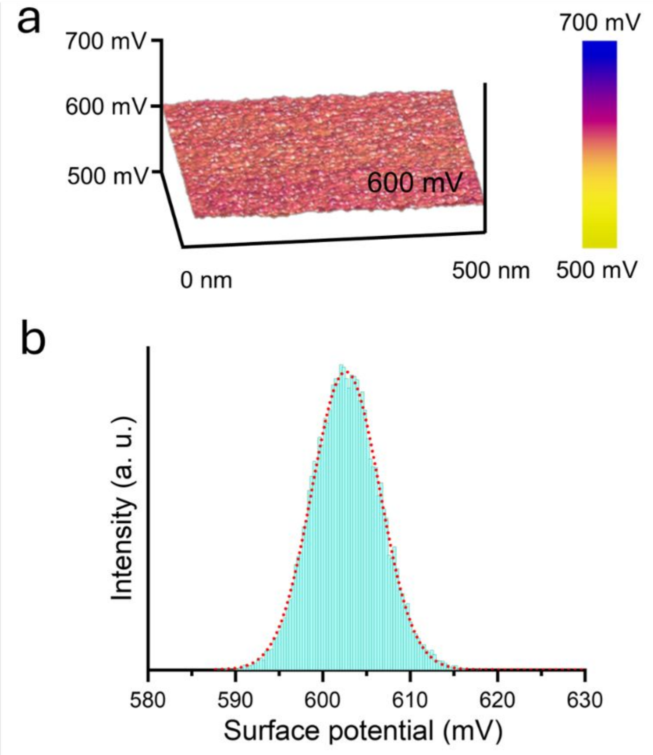

Figure S18. Contact potential difference calibration using highly oriented pyrolytic graphite (HOPG) as a reference standard. (a) The measurement surface potential mapping was performed with a single-crystal diamond-based conductive AFM tip (AD-2.8-AS) to quantify the work function values of the tip. (b) The Gaussian distribution of the surface potential of HOPG substrate.

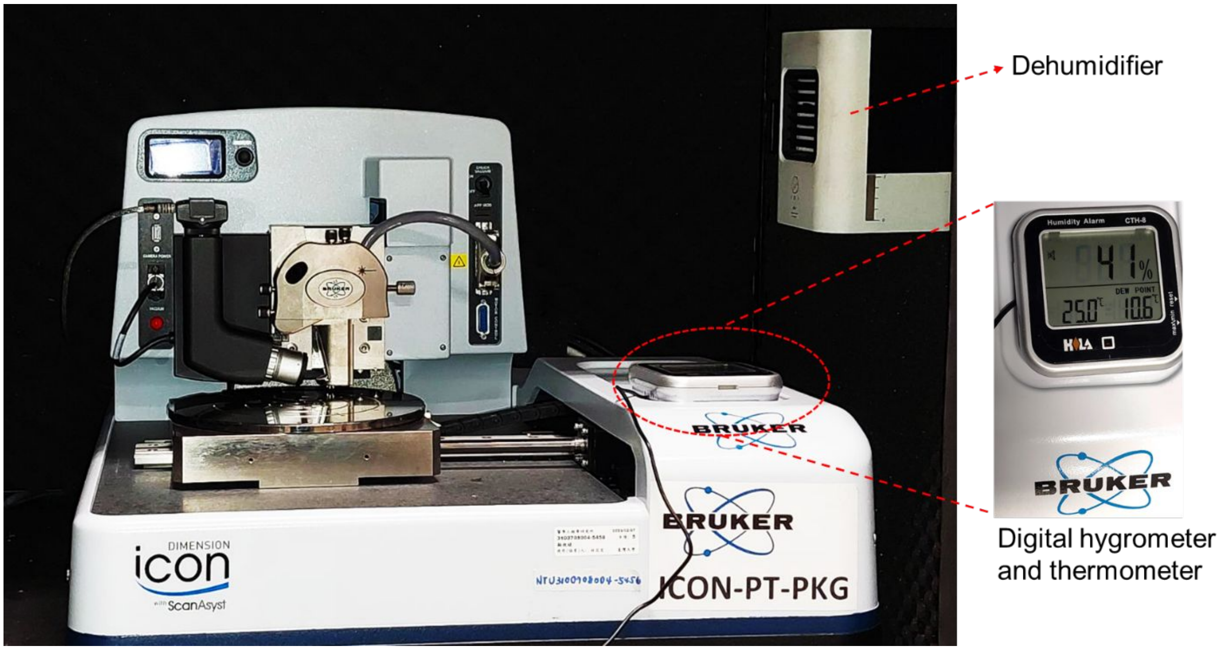

Figure S19. Experimental setup for characterizing the chiral amino acid-modified CuO TENG surface before and after the contact electrification, under controlled environmental conditions using KPFM. The setup includes a Bruker Dimension Icon atomic force microscope (AFM) system with integrated scanning probe capabilities for precise mechanical actuation and electrical measurements. A dehumidifier is used to control the ambient humidity levels during testing, while a digital hygrometer and thermometer (showing 40±1% humidity at 25.0±1°C) monitor the environmental conditions in real-time. This controlled environment setup enables systematic investigation and ensures reproducible measurements of the chiral amino acid-coated surfaces.

Reference

(1) Akerlöf, G. Dielectric Constants of Some Organic Solvent–Water Mixtures at Various Temperatures. *J. Am. Chem. Soc.* **1932**, *54* (11), 4125–4139.

(2) Bowden, N. A.; Sanders, J. P. M.; Bruins, M. E. Solubility of the Proteinogenic α-Amino Acids in Water, Ethanol, and Ethanol–Water Mixtures. *J. Chem. Eng. Data* **2018**, *63* (3), 488–497.

(3) Dakin, H. D. On amino acids. *Biochem. J.* **1918**, *12*, 290–292.

(4) Lammens, T. M.; Potting, J.; Sanders, J. P. M.; De Boer, I. J. M. Environmental Comparison of Biobased Chemicals from Glutamic Acid with Their Petrochemical Equivalents. *Environ. Sci. Technol.* **2011**, *45*, 8521–8528.

(5) Sawamura, S.; Kunimasa, N. High-Pressure Solubility of L-Methionine in Water. *J. Solution Chem.* **2014**, *43*, 1810–1815.

(6) Lu, J.; Lin, Q.; Rohani, S.; Li, Z. Solubility of L-Phenylalanine Anhydrous and Monohydrate Forms: Experimental Measurements and Predictions. *J. Chem. Eng. Data* **2012**, *57* (5), 1492–1498.

(7) Reichardt, C.; Welton, T. *Solvents and Solvent Effects in Organic Chemistry*; John Wiley & Sons: Hoboken, NJ, **2010**.

(8) McGarry, J. Correlation and prediction of the vapor pressures of pure liquids. *Ind. Eng. Chem. Process Des. Dev.* **1983**, *22* (2), 313–319.

(9) Kulkarni, S.; Joshi, M. S. Design and Analysis of Shielded Vertically Stacked Ring Resonator as Complex Permittivity Sensor for Petroleum Oils. *IEEE Trans. Microw. Theory Tech.* **2015**, 63 (8), 2411–2417.

(10) Stoppa, A.; Nazet, A.; Buchner, R.; Thoman, A.; Walther, M. Dielectric Response and Collective Dynamics of Acetonitrile. *J. Mol. Liq.* **2015**, 212, 963–968.

(11) Ewing, M. B.; Sanchez Ochoa, J. C. Vapor Pressures of Acetonitrile Determined by Comparative Ebulliometry. *J. Chem. Eng. Data* **2004**, 49, 486–491.

(12) Kneisl, P.; Weidlich, E.; Wilhelm, E. Vapor pressure, liquid density, and the latent heat of vaporization as functions of temperature for four dipolar aprotic solvents, *J. Chem. Eng. Data* **1987**, 32 (3), 307–311.

(13) Mohsen-Nia, M.; Amiri, H.; Jazi, B. Dielectric Constants of Water, Methanol, Ethanol, Butanol and Acetone: Measurement and Computational Study. *J. Solution Chem.* **2010**, 39, 701–708.

(14) National Institute of Standards and Technology (NIST). *WebBook Chemistry Database*; https://webbook.nist.gov/chemistry/

(15) Lin, S.; Xu, L.; Wang, A. C.; Wang, Z. L. Quantifying Electron-Transfer in Liquid-Solid Contact Electrification and the Formation of Electric Double-Layer. *Nat. Commun.* **2020**, 11, 399.

(16) Sun, M.; Lu, Q.; Wang, Z. L.; Huang, B. Understanding Contact Electrification at Liquid–Solid Interfaces from Surface Electronic Structure. *Nat. Commun.* **2021**, 12, 1752.

(17) Nie, J.; Wang, Z.; Lin, S.; Chen, J.; Wang, Z. L. Probing Contact-Electrification-Induced Electron and Ion Transfers at a Liquid–Solid Interface. *Adv. Mater.* **2020**, 32, 1905696.

(18) Zhan, F.; Li, L.; Wang, S.; Wu, Y.; Wang, Z.; Wang, Z. L. Electron Transfer as a Liquid Droplet Contacting a Polymer Surface. *ACS Nano* **2020**, 14, 17565–17573.

(19) Barlow, S. M.; Raval, R. Complex Organic Molecules at Metal Surfaces: Bonding, Organisation and Chirality. *Surf. Sci. Rep.* **2003**, 50, 201–341.

(20) Zaera, F. Chiral Modification of Solid Surfaces: A Molecular View. *J. Phys. Chem. C* **2008**, 112, 16196–16203.

(21) Ernst, K.-H. Molecular Chirality at Surfaces. *Phys. Status Solidi B* **2012**, 249, 2057–2088.

(22) Wang, Z.; Chen, J.; Zhang, X.; Wang, A. C.; Wu, C.; Xu, C.; Wang, Z. L. Contact-Electro-Catalysis for the Degradation of Organic Pollutants Using Pristine Dielectric Powders. *Nat. Commun.* **2022**, 13, 130.

(23) Li, S.; Wang, H.; Yu, J.; Wu, Z.; Chen, X.; Wang, Z. Contributions of Different Functional Groups to Contact Electrification of Polymers. *Adv. Mater.* **2020**, 32, 2001307.

(24) Marcus, R. A. Electron Transfer Reactions in Chemistry: Theory and Experiment (Nobel Lecture). *Angew. Chem. Int. Ed.* **1993**, 32, 1111–1121.

(25) Barbara, P. F.; Meyer, T. J.; Ratner, M. A. Contemporary Issues in Electron Transfer Research. *J. Phys. Chem.* **1996**, 100, 13148–13168.

(26) Zhou, Q.; Pan, J.; Deng, S.; Xia, F.; Kim, T. Triboelectric Nanogenerator-Based Sensor Systems for Chemical or Biological Detection. *Adv. Mater.* **2021**, 33, 2008276.

(27) Reichardt, C. Solvatochromic Dyes as Solvent Polarity Indicators. *Chem. Rev.* **1994**, *94*, 2319–23580.

(28) Kang, Y.; Hu, T.; Wang, Y.; He, K.; Wang, Z.; Hora, Y.; et al. Nanoconfinement Enabled Non-Covalently Decorated MXene Membranes for Ion-Sieving. *Nat. Commun.* **2023**, *14*, 6121.

(29) Zaera, F. Chiral modification of solid surfaces: a molecular view. *J. Phys. Chem. C* **2008**, *112*, 16196–16203.

(30) Ernst, K.-H. Molecular chirality at surfaces. *Phys. Status Solidi B* **2012**, *249*, 2057–2088.

(31) Barlow, S. M.; Raval, R. Complex organic molecules at metal surfaces: bonding, organisation and chirality. *Surf. Sci. Rep.* **2003**, *50*, 201–341.

(32) Fleming, G. J.; Adib, K.; Rodriguez, J. A.; Barteau, M. A.; White, J. M. The adsorption and reactions of the amino acid proline on TiO_2(110) surfaces. *Surf. Sci.* **2008**, *602*, 2029–2038.

(33) Schiffrin, A.; Mercier, L.; Lyons, I.; Wittstock, G.; Shin, G.-Y.; Raval, R. Zwitterionic self-assembly of L-methionine nanogratings on the Ag(111) surface. *Proc. Natl Acad. Sci. USA* **2007**, *104*, 5279–5284.

(34) Uvdal, K.; Bodo, P.; Liedberg, B. L-cysteine adsorbed on gold and copper: an x-ray photoelectron spectroscopy study. *J. Colloid Interface Sci.* **1992**, *149*, 162–173.

(35) Peluso, P.; Chankvetadze, B.; Cossu, S. Chiral recognition in the domain of molecular chirality: an overview. *Chem. Rev.* **2022**, *122*, 13235–13400.

(36) Vazdar, M.; Heyda, J.; Mason, P. E.; Tesei, G.; Allolio, C.; Lund, M.; Jungwirth, P. Arginine "magic": Guanidinium like-charge ion pairing from aqueous salts to cell penetrating peptides. *Acc. Chem. Res.* 2018, 51 (6), 1455–1464.

(37) Love, J. C.; Estroff, L. A.; Kriebel, J. K.; Nuzzo, R. G.; Whitesides, G. M. Self-assembled monolayers of thiolates on metals as a form of nanotechnology. *Chem. Rev.* **2005**, *105*, 1103–1170.

(38) Castner, D. G.; Hinds, K.; Grainger, D. W. X-ray photoelectron spectroscopy sulfur 2p study of organic thiol and disulfide binding interactions with gold surfaces. *Langmuir* **1996**, *12*, 5083–5086.

(39) Lorenzo, M. O.; Thornton, G.; Haq, S.; Raval, R. Creating chiral surfaces for enantioselective heterogeneous catalysis: R,R-tartaric acid on Cu(110). *J. Phys. Chem. B* **1999**, *103*, 10661–10668.

(40) Rankin, R. B.; Sholl, D. S. Assessment of heterochiral and homochiral glycine adlayers on Cu(110). *J. Phys. Chem. B* **2005**, *109*, 16764–16773.

(41) Ghosal, S.; Hemminger, J. C.; Bluhm, H.; Mun, B. S.; Hebenstreit, E. L. D. Electron spectroscopy of aqueous solution interfaces reveals surface enhancement of halides. *Science* **2005**, *307*, 563–566.

(42) Biesinger, M. C.; Payne, B. P.; Grosvenor, A. P.; Lau, L. W. M.; Gerson, A. R.; Smart, R. S. C. Resolving surface chemical states in XPS analysis of first row transition metals, oxides and hydroxides: Cr, Mn, Fe, Co and Ni. *Appl. Surf. Sci.* **2011**, *257*, 2717–2730.

(43) Arnolds, H.; Bonn, M. Ultrafast surface vibrational dynamics. *Surf. Sci. Rep.* **2010**, *65*, 45–66.

(44) Humblot, V.; Lorenzo, M. O.; Baddeley, C. J.; Haq, S.; Raval, R. Local and global chirality at surfaces: succinic acid versus tartaric acid on Cu(110). *J. Am. Chem. Soc.* **2004**, *126*, 6460–6469.

(45) Zong, Y.; Zhang, C.; Cao, H. Chiral functionalization of solid surfaces with amino acid derivatives: diazonium grafting regulated by enantioselective processes. *Dalton Trans*. **2022**, 51 (39), 14906–14911.

(46) Mallat, T.; Orglmeister, E.; Baiker, A. Asymmetric catalysis at chiral metal surfaces. *Chem. Rev.* **2007**, *107*, 4863–4890.

(47) Li, Y.; Deng, K.; Shen, C.; Liang, X.; Zeng, Z.; Liu, L.; Xu, X. Enantiomeric virus-inspired oncolytic particles for efficient antitumor immunotherapy. *ACS Nano* **2023**, *17*, 17320–17331.

(48) Liu, Y.; Wu, Z.; Kollipara, P. S.; Montellano, R.; Sharma, K.; Zheng, Y. Label-free ultrasensitive detection of abnormal chiral metabolites in diabetes. *ACS Nano* **2021**, *15*, 6448–6456.

(49) Skvortsova, A.; Han, J. H.; Tosovska, A.; Bainova, P.; Kim, R. M.; Burtsev, V.; Erzina, M.; Fitl, P.; Urbanova, M.; Svorcik, V.; Ha, I. H. Enantioselective molecular detection by surface enhanced Raman scattering at chiral gold helicoids on grating surfaces. *ACS Appl. Mater. Interfaces* **2024**, *16*, 48526–48535.

(50) Wu, F.; Tian, Y.; Luan, X.; Lv, X.; Li, F.; Xu, G.; Niu, W. Synthesis of chiral Au nanocrystals with precise homochiral facets for enantioselective surface chemistry. *Nano Lett.* **2022**, *22*, 2915–2922.

(51) Yang, X. L.; Yang, Z. Y.; Shao, R.; Guan, R. F.; Dong, S. L.; Xie, M. H. Chiral MOF derived wearable logic sensor for intuitive discrimination of physiologically active enantiomer. *Adv. Mater.* **2023**, *35*, 2304046.

(52) Arabi, M.; Ostovan, A.; Wang, Y.; Mei, R.; Fu, L.; Li, J.; Wang, X.; Chen, L. Chiral molecular imprinting-based SERS detection strategy for absolute enantiomeric discrimination. *Nat. Commun.* **2022**, *13*, 5757.

(53) Miliutina, E.; Shilenko, V.; Burtsev, V.; Petkevich, A.; Elashnikov, R.; Buravets, V.; Kolska, Z.; Kohout, M.; Svorcik, V.; Lyutakov, O. Homochiral optical fibers coated with nanoscale films of metal–organic frameworks and double-plasmonic Ag and Au for in situ enantioselective detection. *ACS Appl. Nano Mater.* **2024**, *7*, 9210–9217.

(54) Xu, J.; Jian, X.; Guo, J.; Zhao, J.; Tang, J.; Zhao, Y.; Xu, J.; Gao, Z.; Song, Y. Y. Selective SERS identification and quantification of glucose enantiomers on homochiral MOF-based enzyme-free nanoreactors. *Chem. Eng. J.* **2023**, *459*, 141650.

(55) Liu, J. Z.; Chai, X. Y.; Huang, J.; Li, R. S.; Li, C. M.; Ling, J.; Cao, Q. E.; Huang, C. Z. Chiral assembly of perovskite nanocrystals: sensitive discrimination of amino acid enantiomers. *Anal. Chem.* **2024**, *96*, 4282–4289.
